# *Ruta montana* L. from Morocco: comprehensive phytochemical analysis and exploration of its antioxidant, antimicrobial, anti-inflammatory and analgesic properties

**DOI:** 10.3389/fchem.2025.1614984

**Published:** 2025-06-20

**Authors:** Mohamed El Ouardi, Aziz Drioiche, Imane Tagnaout, Aicha Benouahi, Omkulthom AL kamaly, Abdelaaty Abdelaziz Shahat, El Makhoukhi Fadoua, Handaq Nadia, Sevser Sahpaz, Touriya Zair, Mohamed Alaoui Belghiti

**Affiliations:** ^1^ Laboratory of Spectroscopy, Molecular Modelling, Materials, Nanomaterial, Water and Environment, CERNE2D, Faculty of Science, Mohammed V, University in Rabat, Rabat, Morocco; ^2^ Research Team of Chemistry of Bioactive Molecules and the Environment, Laboratory of Innovative Materials and Biotechnology of Natural Resources, Faculty of Sciences, Moulay Ismaïl University, Meknes, Morocco; ^3^ Higher Institute of Nursing Professions and Health Techniques of Fez, Regional Health Directorate Fez-Meknes, EL Ghassani Hospital, Fez, Morocco; ^4^ Department of Pharmaceutical Sciences, College of Pharmacy, Princess Nourah bint Abdulrahman University, Riyadh, Saudi Arabia; ^5^ Pharmacognosy Department, College of Pharmacy, King Saud University, Riyadh, Saudi Arabia; ^6^ University Lille, University of Liège, University of Picardie Jules Verne, JUNIA, UMRT 1158 BioEcoAgro, Specialized Metabolites of Plant Origin, Lille, France

**Keywords:** *Ruta montana*, 2-undecanone, rosmarinic acid 3′-glucoside, p-Coumaroylquinic acid, quercitrin, antioxidant, antimicrobial, anti-inflammatory

## Abstract

*Ruta montana* L., a medicinal plant native to Morocco's Middle Atlas region, has been traditionally used for its therapeutic properties. This study aims to investigate its phytochemical composition and evaluate its biological and pharmacological activities, with a focus on its essential oil (EO) and phenolic extracts. The essential oil was extracted via hydrodistillation and analyzed using GC-MS to determine its chemical composition. Aqueous, hydro-ethanolic, and hydro-methanolic extracts were prepared and analyzed for their polyphenol, flavonoid, and tannin content using spectrophotometric methods and HPLC/UV ESI-MS. Antimicrobial activity was assessed using minimum inhibitory concentration (MIC) assays, while antioxidant potential was evaluated using the DPPH radical scavenging method. Analgesic and anti-inflammatory effects were tested using abdominal writhing and edema inhibition models, respectively. Subacute toxicity was assessed by monitoring organ weights and biochemical parameters in treated animals. The EO was predominantly composed of 2-undecanone (81.16%) and decyl propanoate (9.33%). Phenolic extracts were rich in rosmarinic acid 3′-glucoside, p-coumaroylquinic acid, quercitrin, ferulic acid, and embelin. The EO exhibited strong antimicrobial activity (MIC = 2.34–37.5 mg/mL), particularly against *Aspergillus niger*, and significant analgesic effects (44.55% reduction in abdominal writhing at 0.2 mL), outperforming the aqueous extract (23.37%). Phenolic extracts demonstrated notable antioxidant activity (IC_50_ = 117.24 μg/mL in DPPH), while the EO showed moderate antioxidant potential (IC_50_ = 29.42 μg/mL; BHT = 1.62 μg/mL). Anti-inflammatory assays revealed that both the EO (71% inhibition at 0.2 mL) and aqueous extract (79% inhibition at 300 mg/kg) were comparable to indomethacin. Subacute toxicity tests indicated no significant organ weight changes, although slight increases in hepatic AST (91.33 U/L) and creatinine (2.36 mg/L) were observed at higher doses. These findings highlight *R. montana's* potential as a natural source of antioxidant, antimicrobial, and anti-inflammatory agents. The EO, in particular, shows promise as a therapeutic alternative. However, further studies are needed to evaluate its long-term safety and efficacy. *R. montana* demonstrates significant pharmacological potential, particularly its essential oil, which warrants further investigation for therapeutic applications.

## 1 Introduction

The discovery of antibiotics was the miracle of the 20th century, significantly reducing mortality associated with infectious diseases and improving the health and wellbeing of humanity. Today, however, this effectiveness is being challenged due to the excessive use of antibiotics aimed at addressing numerous human illnesses. Alas, this misuse has extended to other fields such as agriculture and livestock farming, thereby contributing to the residual contamination of food and, subsequently, the natural evolution of antibiotic resistance in pathogenic microbial strains (Okaiyeto et al., 2024). As a result, it has become increasingly difficult to treat infections that were once curable, leading to situations of therapeutic impasse (Touat et al., 2019). The phenomenon of antibiotic resistance has now become entrenched in our daily lives and represents a global concern. To address this issue, extensive research has been conducted in recent years to develop new antimicrobial agents of natural origin and, consequently, produce safe food products (Lucera et al., 2012; Mendonca et al., 2018; Lucera et al., 2012).

On the other hand, interest in natural antioxidants continues to grow among consumers, healthcare professionals, and food scientists, owing to their ability to protect food from oxidative deterioration and shield the body from the harmful effects of oxidative stress (García-Caparrós et al., 2021). Consequently, several natural antioxidants have been isolated for use as pharmaceutical and nutraceutical products, as well as food preservatives (Gutiérrez-del-Río et al., 2018; Lourenço et al., 2019; Fernandes et al., 2020; Teixeira et al., 2022). This popular trend has spurred numerous scientific studies aimed at exploring the chemical composition and biological properties of extracts and essential oils (EOs) derived from novel plant sources. In this context, certain species of *Ruta* (Rutaceae) have been investigated for their chemical components and pharmacological activities. Species of the Rutaceae family are renowned for their economic importance, including their citrus fruits and cultivated essential oils (Groppo et al., 2022).

The genus *Ruta* comprises around sixty species, some of which are found in the Mediterranean region (Hammiche and Azzouz, 2013). Among these, *Ruta montana* is widely distributed geographically, particularly in Morocco, Algeria, Tunisia, Portugal, Greece, and Turkey (Mohammedi et al., 2019). It is a semi-shrub with evergreen foliage, ranging from 40 to 60 cm in height, highly branched, and woody at the base. Its leaves are fine and triangular in shape, while its small yellow flowers feature two whorls of stamens and are bisexual. Its fruits are capsules with four rounded lobes. The essential oil (EO) contained in large pockets housing secretory glands gives *R. montana* a pungent odor. *R. montana* is commonly referred to as “fidjel” in Arabic and “aourmi” in Berber (Bellakhdar, 1997).

In traditional Moroccan medicine, decoctions and infusions of mountain rue are used for the treatment of diabetes (Tahraoui et al., 2007). An ethnobotanical study conducted among herbalists revealed that *R. montana* L. has a high usage value in southwestern Morocco, with an index of 7 (El-Ghazouani et al., 2021). Its decoction is frequently used for treating oral and dental diseases, as well as for gum rinsing, by the population of the Middle Atlas (Najem et al., 2020). Another ethnobotanical study cited the use of infusions and decoctions of *R. montana* flowers for treating bronchial congestion and asthma in the central Middle Atlas region of Morocco (Najem et al., 2021). Bencheikh et al. (2021) reported the use of decoctions of this plant in the traditional treatment of kidney diseases. This species is also used in Algeria to treat digestive disorders (Adli et al., 2021), alleviate toothaches and joint pain, and facilitate difficult childbirths (Hammiche and Azzouz, 2013; Miara et al., 2019; Adli et al., 2021). Benkhaira et al. (2022) documented the use of *R. montana* for treating diabetic, digestive, respiratory, neurological, and gynecological ailments in North Africa.

On a scientific level, several *in vitro* and *in vivo* studies have demonstrated the various biological potentials of *R. montana*, including antibacterial properties (Zellagui et al., 2012; Mohammedi et al., 2019; Benali et al., 2020b), antifungal properties (Drioiche et al., 2020a; Slougui et al., 2023; Bouhenna et al., 2024), antioxidant properties (Kambouche et al., 2008; Benali et al., 2020a), antihypertensive effects (El-Ouady and Eddouks, 2021), antidiabetic activity (Farid et al., 2017), anti-acetylcholinesterase activity (Khadhri et al., 2017), anticancer properties (Kara Ali et al., 2016), antifertility effects (Merghem et al., 2017), as well as insecticidal and larvicidal properties (Boutoumi et al., 2009; Bouzeraa et al., 2019; Bouabida and Dris, 2022). Despite the numerous therapeutic benefits of *R. montana*, it can easily become toxic if improperly dosed. An ethnobotanical study conducted in northern Morocco cited this plant as toxic, causing digestive and neurological disorders (Kharchoufa et al., 2021). Previous studies have highlighted the richness of *R. montana* in various phytochemical compounds, including alkaloids, coumarins, flavonoids, tannins, and volatile compounds (Daoudi et al., 2016; Bouhenna et al., 2024).

Although *R. montana* has been the subject of several research studies regarding its chemical composition and biological activities, relevant data on Mountain Rue collected from Morocco are often scattered and fragmented. Furthermore, few scientific studies have investigated its phenolic profile, and many pharmacological activities, such as anti-inflammatory and analgesic properties, remain unexplored. Therefore, the aim of this work is to determine the chemical composition of the essential oil (EO) and the phenolic profile of *R. montana* extracts, as well as to evaluate their antioxidant and antimicrobial effects. Additionally, it seeks to examine, for the first time, the anti-inflammatory and analgesic activities, as well as to assess the subacute toxicity of the extracts and the EO.

## 2 Materials and methods

### 2.1 Plant material

**FIGURE 1:**
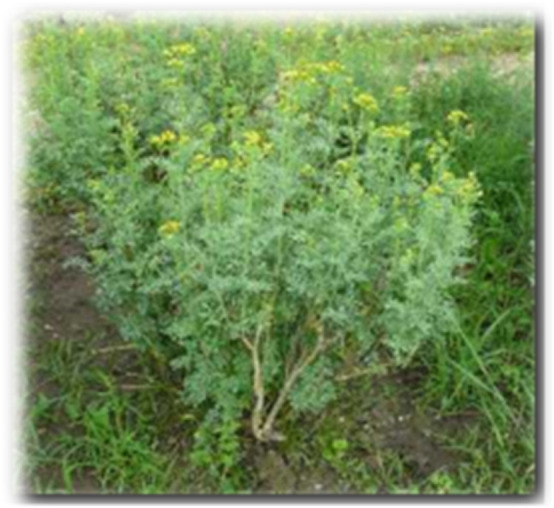
*R. montana* L. (El Ouardi Mohamed and Touriya Zair, 2024).

The aerial parts of *R. montana* were collected in June 2024 from wild populations in the Boulemane region, specifically in the Municipality of Guigou (Figure 1). The harvesting site, the harvested plant part, and its source are detailed in Supplementary Table S1. Botanical identification was carried out through a meticulous morphological analysis of the specimens, comparing them with herbarium material and consulting botanical literature. After the plant was identified by a botanist, represented by Professor Amina Bari from the Biology Department of the Faculty of Sciences (FSDM) at USMBA in Fez, the samples were deposited in the herbarium of the Biology Department under the reference number LRM00252024. After harvesting, the collected plant material was dried to preserve it. The aerial parts were spread out in a single layer and dried at room temperature (protected from direct sunlight) in dry, clean, and well-ventilated areas to minimize the degradation of bioactive substances. The drying process was conducted at a temperature of 25°C for 2 weeks, until the material was completely dehydrated. Each sample of dried plant material was then transferred into a labeled paper bag and stored in a cool, dark place at room temperature for future analysis.

### 2.2 Microbiological materials

In this study, the antimicrobial activity of the EO and extracts from the flowering tops of *R. montana* was evaluated against five bacterial strains and five fungal strains. These microorganisms are contaminants and pathogens widely identified in various human pathologies (Table 1). All strains were successively revitalized in Mueller-Hinton and Sabouraud broths, subcultured, and stored in a 20% glycerol stock at −80°C.

**TABLE 1:** List of tested bacterial and fungal strains used for antimicrobial tests.

		Strains	Abbreviations
Bacteria	Gram-negative bacilli	*Enterobacter cloacae*	*E. cloacae*
		*Klebsiella pneumoniae*	*K. pneumoniae*
		*Escherichia coli*	*E. coli sauvage*
	Gram-positive cocci	*Staphylococcus aureus*	*S. aureus*
		*Staphylococcus epidermidis*	*S.epidermidis*
Fungi	Yeasts	*Candida albicans*	*C.albicans*
		*Candida dubliniensis*	*C. dubliniensis*
		*Candida tropicalis*	*C. tropicalis*
		*Candida parapsilosis*	*C. parapsilosis*
	Molds	*Aspergillus niger*	*A.niger*

### 2.3 Animal material

In this study, male and female Swiss albino mice (weighing between 30 and 35 g) as well as Wistar rats (weighing between 200 and 250 g) were used as animal models. The animals were initially weighed and then housed individually in polypropylene plastic cages in a controlled temperature environment, with a 12-h light/dark cycle and unlimited access to food and water. All experimental procedures and protocols were meticulously conducted in strict adherence to ethical guidelines and regulations to ensure animal welfare, with the study being reviewed and approved by the Institutional Animal Care and Use Committee (IACUC) of the Faculty of Sciences Dhar El Mehraz, Sidi Mohamed Ben Abdallah University, Fès, Morocco, under reference #04/2019/LBEAS, in accordance with institutional ethical standards and the European Economic Community (EEC) Directive 86/609/EEC (Council Directive 86/609/EEC of 24 November, 1986).

### 2.4 Quality control of plant material

#### 2.4.1 Moisture content determination

The procedure used to determine the moisture content complies with the AFNOR standard (AFNOR Editions, 1985; Saidi et al., 2023). A quantity of 5 g of plant sample was weighed in crucibles that had been previously dried and tared. The crucibles containing the plant material were then placed in an oven at a temperature between 103°C and 105°C for 24 h. After this period, they were cooled in a desiccator and weighed. The moisture content was calculated using the following Formula 1:

$$TH\%=\left(\frac{m_{0}-m_{1}}{m_{0}}\right)\times 100$$ (1)

where:

m_0_: Initial mass of the plant material (in grams)

m_1_: Mass after drying (in grams)

The result is expressed as a percentage of dry matter.

#### 2.4.2 Determination of pH

The method involves mixing 2 g of ground sample with 10 mL of warm distilled water. After stirring, the cooled mixture is filtered. The pH meter electrode is then immersed in a volume of the filtrate to record the pH value (Saidi et al., 2023).

#### 2.4.3 Determination of titrable acidity

Titrable acidity corresponds to the sum of free mineral and organic acids. The principle of this method is based on an acid-base titration using a NaOH solution. A quantity of 2 g of the ground plant sample was added to 100 mL of boiling distilled water. After 15 min of stirring, the mixture was filtered and then titrated with a NaOH solution (N = 0.01), in the presence of a few drops of phenolphthalein, until a color change was observed, resulting in a persistent pink color lasting approximately 30 s. The recorded titration volume is converted into citric acid equivalent by multiplying it by a factor obtained from the following calculation (Equation 2) (AFNOR Editions, 1985; Saidi et al., 2023).

$$Titratable\;acidity\;\left(\%\right)=Dilution\;factor*\;\frac{weight\;of\;acid\;equiv.*NaOH\;normality*titration\;vol.\left(mL\right)}{sample\;weight\;\left(g\right)}$$ (2)

#### 2.4.4 Mineral matter (ash) and organic matter content

The ash content corresponds to the mineral matter remaining after the destruction of organic matter by incineration at high temperatures in a furnace. A quantity of 5 g of ground sample is placed in a muffle furnace at a temperature of 550°C until the complete destruction of all carbonaceous particles and the attainment of whitish ash of constant weight (AFNOR Editions, 1972). The organic matter content was calculated using the following Formula 3 (Saidi et al., 2023):

$$MO\%=\left(\frac{M_{1}-M_{2}}{PE}\right)\times 100$$ (3)

where:MO%: Organic matter content.M_1_: Weight of the crucible and the sample before incineration.M_2_: Weight of the crucible and the sample after incineration.PE: Test portion.

The ash content was calculated as follows (Equation 4):

Ash%=100−MO% (4)

#### 2.4.5 Heavy metal analysis: inductively coupled plasma atomic emission spectrometry (ICP-AES)

The analysis of heavy metals in *R. montana* seeds was performed using the Inductively Coupled Plasma Atomic Emission Spectrometry technique, following the standardized mineralization protocol (AFNOR, 1999). This method first involves preparing the sample in liquid form by mixing 0.1 g of plant powder with 3 mL of aqua regia, prepared from 1 mL of nitric acid HNO_3_ (99%) and 2 mLof hydrochloric acid HCl (37%). The mixture is placed in a reflux setup at 200°C for 2 h to ensure the complete dissolution of residual metal particles. After cooling and decantation, the supernatant is collected, filtered through a 0.45 µm membrane, and adjusted to 15 mL with distilled water. The concentrations of heavy metals, including arsenic (As), cadmium (Cd), chromium (Cr), iron (Fe), lead (Pb), antimony (Sb), and titanium (Ti), were determined using the inductively coupled plasma atomic emission spectrometer ICP-AES (Ultima 2 Jobin Yvon) at the Technical Support Unit for Scientific Research (UATRS) laboratory at the CNRST in Rabat (Skujins, 1998; Ailli et al., 2023).

#### 2.4.6 Phytochemical screening

This qualitative analysis aims to identify the primary and secondary metabolites present in the flowering tops of *R. montana*. The tests rely on visual observations of color changes, precipitate formation, and complex formation, while other tests involved examining samples under UV light. The detection of chemical compound groups was carried out according to the protocols described in previous studies, including those by Bekro et al. (2007), Bruneton (2009), Dohou et al. (2003), Mezzoug et al. (2007), Mogode (2005), and N’Guessan et al. (2009).

### 2.5 Extraction and quality control of essential oils

#### 2.5.1 Extraction and determination of essential oil yields

The flowering tops of *R. montana* were hydrodistilled to extract essential oils (EOs). A 2-L flask containing 100 g of the plant material was immersed in 1 L of water and placed in a Clevenger apparatus equipped with a spherical condenser. After 3 h of boiling, the essential oils were distilled off continuously and collected. All of the experiments were carried out in triplets. The essential oils isolated were separated from the aqueous phase under reduced pressure using a graduated cylinder’s separator in hydrodistillation. The oils were dried; using anhydrous sodium sulfate (Na_2_SO_4_), to obtain the completely dried samples. The diluted essential oils were used for Gas chromatography-mass spectrometry (GC-MS) analysis with hexane as a solvent. This dilution made it easier to distinguish and identify volatiles during analysis. Dry essential oils were kept at −4°C in tightly capped amber glass vials after dilution, to preserve their chemical composition until analysis. The yield of EO (%EO) extraction was expressed as the volume of essential oil per mass of plant material (V/M) according to the following Formula 5 (Akrout et al., 2001). The use of hexane as a solvent for GC-MS analysis was chosen due to its high volatility and low interference with the detection of essential oil constituents.

$$EO\%=\left[\frac{V}{M_{0}-\left(M_{0}\times \%MC\right)}\times 10^{4}\right]\mp Ecart-type$$ (5)

where:

MC (%): Moisture content of the plant material (percentage of humidity or water content).

M_0_: Mass of the distilled plant material.

V: Volume of essential oil collected (in ml).

#### 2.5.2 Analysis and identification of the chemical composition of essential oils

The chromatographic analysis of essential oils was performed using a Thermo Electron gas chromatograph (Trace GC Ultra) coupled with a mass spectrometer (Thermo Electron Trace MS system; Polaris Q MS). Fragmentation was carried out by electron impact at an intensity of 70 eV. The chromatograph was equipped with a DB-5 column (5% phenyl-methyl-siloxane) measuring 30 m × 0.25 mm with a film thickness of 0.25 μm. The system included a flame ionization detector (FID) powered by a hydrogen/air gas mixture. The column temperature was programmed to increase at a rate of 4°C/min from 50°C to 200°C, maintaining this temperature for 5 min. The injection mode was split (split ratio: 1/70, flow rate: mL/min), and nitrogen was used as the carrier gas at a flow rate of 1 mL/min. The identification of the chemical composition of essential oils was achieved by comparing their calculated Kovats indices (KI) with those provided by Adams and known reference products in the literature (Davies, 1990; Goodner, 2008; Jennings, 2012). This was further complemented by comparing the indices and mass spectra with various references (Adams, 2007; Linstrom and Mallard, 2001). A cut-off match factor of 80% was used to ensure accurate identification of compounds. Additionally, peak deconvolution was performed using the AMDIS software to resolve overlapping peaks and improve identification accuracy.

### 2.6 Extraction of phenolic compounds

Phenolic compounds are considered multifunctional agents in medicinal chemistry, pharmaceutical development, and various synthetic processes (Rappoport, 2004; Makarem et al., 2019; 2023; Klika et al., 2021). Thus, studying the phenol content in plant extracts can reveal their biological profile (Alara et al., 2021).

In this section, the extraction of phenolic compounds was carried out using decoction and solid-liquid extraction with the aid of a Soxhlet apparatus. The decoction process was performed by adding 30 g of plant powder to 600 mL of distilled water. The resulting mixture was heated and brought to a boil under stirring for 1 h at 80°C. Subsequently, the decanted mixture was filtered under low pressure, and the decoction extract was recovered in powdered form in a glass vial. After drying the extract in an oven at 70°C, the decoction extract was stored in a tinted glass bottle, protected from light. As for the second extraction method, the Soxhlet apparatus was employed. In this process, 30 g of plant powder were placed in a cartridge and brought into contact with extraction solvents, specifically ethanol (70/30) and methanol (70/30) solutions. The extracts were concentrated using a rotary evaporator after several extraction cycles. The extracts were coded in accordance with Supplementary Table S2, which outlines the codification of *R. montana* extracts.

### 2.7 Determination of phenolic compounds

The quantification of total phenols in various extracts was performed using the Folin-Ciocalteu method, as described by Singleton and Rossi (1965). The absorbance was recorded using a UV mini-1240 spectrophotometer set at 760 nm and compared to a blank. A calibration curve was established using gallic acid as a positive control. The results were expressed in milligram gallic acid equivalent per gram of extract (mg GAE/g). Each test was performed in triplicate.

### 2.8 Quantification of flavonoids

The quantification of flavonoids was performed using the method established by Djeridane et al. (2006). The flavonoid content was calculated based on a calibration curve using quercetin as the standard. The results are expressed in milligrams of quercetin equivalent per gram of extract (mg QE/g). Each test was conducted in triplicate.

### 2.9 Determination of condensed tannins

The quantification of condensed tannins was carried out using the vanillin method, as described by Price et al. (1978). In this technique, a vanillin/methanol solution (4% w/v) was mixed with varying quantities of (+)-catechin solution (2 mg/mL) and manually agitated. Each concentration was then placed in a test tube containing 1.5 mL of hydrochloric acid. The reaction mixture was left at room temperature for 20 min. Absorbance was measured at 499 nm using a UV-visible spectrophotometer, with reference to a blank. The condensed tannin content in our samples was calculated using the calibration curve of catechin as the standard (Y = 0.7421X + 0.0318; R^2^ = 0.998). The tannin content is expressed in milligrams of catechin equivalent per gram of extract (mg CE/g).

### 2.10 HPLC/UV ESI-MS analysis of the three extracts of *R. montana*

The analysis of phenolic compounds in the three extracts of *R. montana* was conducted using high-performance liquid chromatography coupled with Q Exactive Plus mass spectrometry, employing electrospray as the molecular ionization method (HPLC/UV-ESI-MS). The analysis was performed on an UltiMate 3000 HPLC system (Thermo Fisher Scientific, Sunnyvale, CA, United States) equipped with an autosampler. The autosampler was set to maintain the samples at *5°C*. The HPLC system utilized a reversed-phase C18 column with a column temperature of *40°C* (Lichro CART, Lichrospher, Merck, Darmstadt, Germany, 250 × 4 mm, ID 5 µm). The mobile phase consisted of solvent A: 0.1% formic acid in water (v/v) and solvent B: 0.1% formic acid in acetonitrile (v/v), with degassing performed ultrasonically. At 20, 25, 26, and 30 min, the gradient composition transitioned from 2% B to 30%, 95%, 2%, and 2% B, respectively. The flow rate was 1 mL/min, and the injection volume was 20 µL. Broadband collision-induced dissociation (bbCID) detection was carried out on a Maxis Impact HD (Bruker Daltonik, Bremen, Germany) following negative electrospray ionization. A diode array detector L-2455 (Merck-Hitachi, Darmstadt, Germany) was also employed for UV detection, with scanning in the range of 190–600 nm and acquisition at three wavelengths: 280, 320, and 360 nm. The parameters used included a capillary voltage of 3,000 V, a drying gas temperature of 200°C, a dry gas flow rate of 8 L/min, a nebulizer gas pressure of 2 bars, and an offset plate voltage of 500 V. Nitrogen was used both as the nebulizing gas and the desolvation gas. The m/z range for MS data acquisition was 100 to 1,500. Data collection and analysis were performed using the Thermo Scientific Chromeleon 7.2 Chromatography Data System (CDS). By examining the mass spectra of the eluted molecules, the eluted compounds were investigated.

### 2.11 Antioxidant activity

#### 2.11.1 Free radical scavenging activity by DPPH• assay

The antiradical effect of the essential oils (EOs) and extracts of *R. montana* was evaluated using the 2,2-diphenyl-1-picrylhydrazyl (DPPH) radical, following the method described by Liu et al. (2009). In test tubes containing absolute ethanol, different concentrations of *R. montana* extract or EO were prepared to reach a total volume of 200 μL. Subsequently, 2.8 mL of an ethanolic DPPH° solution (24 μg/mL, equivalent to a molar concentration of 6.12 × 10^−5^ M) were added to the mixture and incubated for 30 min in the dark. Absorbance was measured at 515 nm using a UV-Vis spectrophotometer. All tests were performed in triplicate. The concentration range used for the extracts was crucial to determine the IC_50_ value, which represents the concentration required to inhibit 50% of the DPPH radicals. This range was systematically varied to ensure accurate calculation of the IC_50_. The reference standard used was butylated hydroxytoluene (BHT) at various concentrations. The results were expressed as the percentage of DPPH• reduction (*PI*%) and calculated using the following Formula 6:

$$PI\%=\frac{A_{0}-A}{A_{0}}\times 100$$ (6)

where:

PI%: Percentage of antioxidant activity.

A_0_: Absorbance of the solution containing only the DPPH• radical solution (negative control).

A: Absorbance of the test samples in the presence of DPPH•.

The half-maximal inhibitory concentration (IC_50_) of the DPPH• radicals, either for BHT or our extracts, was determined from the graph plotting the variation of antioxidant activity as a function of concentration.

#### 2.11.2 Ferric reducing antioxidant power (FRAP) method

The reducing power of the phenolic extracts from *R. montana* to reduce ferric iron (Fe^3+^) present in the potassium ferricyanide complex to ferrous iron (Fe^2+^) was determined using the method described by Oyaizu (1986). The assay involves mixing 1 mL of the plant extract with 2.5 mL of phosphate buffer (0.2 M, pH 6.6) and 2.5 mLof a 1% potassium ferricyanide (K_3_Fe(CN)_6_) solution. The resulting mixture is incubated in a water bath at 50°C for 20 min. Subsequently, 2.5 mL of 10% trichloroacetic acid is added to stop the reaction. The mixture is then centrifuged at 3,000 rpm for 10 min. Finally, 2.5 mL of the supernatant from each concentration is mixed with 2.5 mL of distilled water and 0.5 mL of a 0.1% aqueous FeCl_3_ solution. The absorbance of the reaction medium is measured at 700 nm against a blank prepared similarly, replacing the aqueous extract with distilled water to calibrate the instrument (UV-Vis spectrophotometer). The positive control is represented by a solution of a standard antioxidant, BHA (butylated hydroxyanisole), whose absorbance was measured under the same conditions as the samples. All tests were performed in triplicate. The graph plotting the variation of reducing power as a function of the concentration of BHT or our extracts was used to determine the concentration corresponding to an absorbance of 0.5 (EC_50_).

#### 2.11.3 Total antioxidant capacity

The phosphomolybdenum assay, as described by Khiya et al. (2021), was used to evaluate the total antioxidant capacity of *R. montana* extracts. This assay is based on the reduction of molybdenum Mo^6+^ (VI) to molybdenum Mo^5+^ (V) in the presence of the extracts, resulting in the formation of a green phosphate/Mo^5+^ (V) complex at acidic pH, with maximum absorbance at 695 nm. In a test tube, 3 mL of the reagent solution (0.6 M sulfuric acid, 28 mM sodium phosphate, and 4 mM ammonium molybdate) was added to a volume of the extract. The tubes were shaken and incubated at 95°C for 90 min, then allowed to return to room temperature. After cooling, the absorbance of the solutions was measured at 695 nm. Ascorbic acid was used as the standard. The results are expressed in milligram equivalents of ascorbic acid per gram of extract (mg AAE/g).

### 2.12 Antimicrobial activity

The Minimum Inhibitory Concentration (MIC) was determined using 96-well microplates and the reference microdilution method (Balouiri et al., 2016). MIC is defined as the lowest concentration of essential oil required to completely inhibit the visible growth of the tested microorganism during incubation. A series of dilutions were prepared from a stock solution of the essential oil dissolved in 10% DMSO, resulting in concentrations ranging from 5 to 0.93 × 10^−2^ mg/mL for each essential oil. These dilutions were prepared in a final volume of 100 µL in Sabouraud broth for fungi and Mueller-Hinton medium for bacteria. Subsequently, 100 µL of microbial inoculum was added to each dilution step, with a final concentration of 10^6^ CFU/mL for bacteria or 10^4^ CFU/mL for fungi. Ten microliters of resazurin were added to each well to measure bacterial growth after a 24-h incubation at 37°C. The color change from purple to pink after a second incubation at 37°C for 2 h indicated microbial growth. The MIC value was determined as the lowest concentration that prevented the color change of resazurin. Growth and sterility controls were included in the 11th and 12th wells, respectively. This test was performed twice for the essential oil. For comparison, 250 mg of terbinafine, a standard antifungal agent used in the study, was ground and dissolved in 2 mL of 10% DMSO. The Minimum Bactericidal Concentration (MBC) and Minimum Fungicidal Concentration (MFC) were determined by transferring 10 µL from wells showing no visible growth onto Mueller-Hinton agar (for bacteria) or Sabouraud broth (for fungi) and incubating for 24 h at 37°C. The lowest concentration of the sample that resulted in a 99.99% reduction in CFU/mL compared to the control was designated as the MBC or MFC. Additionally, the MBC/MIC or MFC/MIC ratio was calculated for each extract to evaluate its antimicrobial potency. If the ratio was less than 4, the essential oil was considered bactericidal/fungicidal, while a ratio greater than 4 indicated a bacteriostatic/fungistatic effect (Bissel et al., 2014). The biological assays were conducted in accordance with globally recognized standardized protocols, following the guidelines of the CLSI (Clinical and Laboratory Standards Institute) for antimicrobial testing.

### 2.13 Anti-inflammatory activity

The anti-inflammatory potential of each extract was evaluated based on Winter et al.’s method (1962) (Winter et al., 1962), with certain modifications. This experiment was repeated twice. Carrageenan at a concentration of 1% was dissolved in saline solution and used as the edema-inducing agent. A total of 35 rats were randomly allocated into 7 groups, each consisting of 5 rats. The difference in footpad circumferences before and after carrageenan injection (Sigma-Aldrich, Ref. 3B-C1804, Barcelona, Spain) was measured in millimeters (mm) at 3, 4, 5, and 6 h using calipers (Küpeli and Yesilada, 2007). The inhibitory activity of each treatment was calculated using the following Formula 7:

$$Percent\;inhibition=100\;\left[1-\left(\frac{a-x}{b-y}\right)\right]$$ (7)

where:

a: Mean paw volume after carrageenan administration.

x: Mean paw volume before the injection.b: Mean paw volume of control rats after the injection.

y: Mean paw volume of control rats before the injection.

This formula quantifies the anti-inflammatory effect by comparing the changes in paw volume between treated and control groups over time.

### 2.14 Antinociceptive activity

The antinociceptive effect of each extract was assessed by counting the number of writhes induced in mice following the intraperitoneal injection of a 0.7% acetic acid solution (10 mL/kg). The tested extracts were administered orally to the mice 1 h prior to the acetic acid injection. The number of writhes was recorded for 30 min after the acetic acid injection (Hernández-Pérez and Rabanal, 2002). This experiment was repeated twice. The percentage inhibition (*PI%*) of the writhes was calculated using the following Formula 8:

$$PI\left(\%\right)=\frac{\left(NC-NT\right)}{NC}*100$$ (8)

where:

NC: Number of writhes in the negative control group.

NT: Number of writhes in the tested groups.

This formula quantifies the antinociceptive effect by comparing the writhes in treated groups to those in the control group, providing a measure of the extract’s ability to reduce pain-induced behavior.

### 2.15 Statistical analysis

The results were expressed as mean values ± standard error of the mean (SEM) to ensure accurate representation of data variability. Statistical analyses were performed using one-way analysis of variance (ANOVA), followed by Tukey’s *post hoc* test to determine significant differences between groups. All analyses were conducted using GraphPad Prism 9 (version 9.5.1, San Diego, CA, United States), a widely recognized statistical software for biological and pharmacological research. A significance threshold of *p < 0.05* was applied to establish statistical relevance, ensuring that only robust and meaningful differences were considered in the interpretation of the results. Correlations between phenolic compound content and antioxidant activities were examined using R software (version 4.4.2).

## 3 Results and discussion

### 3.1 Quality control of plant material

#### 3.1.1 Determination of moisture content, pH, acidity, ash, and organic matter

The results of the quality control analysis for the flowering tops of *R. montana* are presented in Table 2.

**TABLE 2:** Analysis and quality control of flowering tops of *R. montana*.

Moisture content (%)	pH	Acidity	Ash	Organic matter
12.14 ± 0.01	5.11 ± 0.01	2.38 ± 0.25	11.77 ± 0.21	88.23 ± 0.21

The moisture content, defined as the amount of water contained within plant cells, is a crucial parameter that influences the quality, shelf life, and therapeutic efficacy of plant material, as well as its susceptibility to microbial contamination. In this study, the moisture content of the powdered flowering tops of *R. montana*, determined using the oven-drying method, was approximately 12.141%, a value slightly above 10%, which is optimal for medium-term storage of the powder during this study. The pH of the plant extract was slightly acidic (5.11), classifying *R. montana* among acidophilic plants that do not tolerate limestone, a characteristic that enhances its capacity for iron absorption, as confirmed by ICP analysis results. Titratable acidity, a parameter ensuring the conformity of the consumed product to specific requirements such as appearance, texture, and taste, was measured at 2.38 ± 0.251 for the studied plant. Additionally, the ash or mineral content, an important indicator of the quality and purity of the plant extract, was found to be 11.77% for the flowering tops of *R. montana*, a value comparable to those reported for *R. graveolens* leaves (11.8%) (Dhale et al., 2010) and *R. angustifolia* leaves (10.75%) (Wahyuni et al., 2023). These findings collectively underscore the quality and specific characteristics of *R. montana*, supporting its potential suitability for therapeutic applications.

#### 3.1.2 Assessment of heavy metal

The presence of certain heavy metals in the environment, which can be absorbed by plants, poses a potential toxicity risk, making the determination of heavy metal content an essential test to ensure the safety and quality of plant materials. In this study, we evaluated the concentrations of eight heavy metals—chromium (Cr), antimony (Sb), arsenic (As), lead (Pb), cadmium (Cd), iron (Fe), copper (Cu), and titanium (Ti)—using Atomic Emission Spectrometry. As shown in Table 3, the flowering tops of *R. montana* exhibited a high iron content of 0.8909 mg/L, while copper was detected at a moderately low concentration of approximately 0.0074 mg/L. Notably, the concentrations of all other detected heavy metals fell within the permissible ranges established by FAO/WHO regulatory standards. These findings indicate that the studied plant can be safely consumed directly, used as an ingredient in food processing, or repackaged as needed without posing a risk to human health.

**TABLE 3:** Concentration of heavy metals (mg/L) (ICP).

Species	Chromium (Cr)	Antimony (Sb)	Arsenic (As)	Lead (Pb)	Cadmium (Cd)	Iron (Fe)	Copper (Cu)	Titanium (Ti)
*R. montana*	0.0021	0.0020	0.0078	0.0300	<0.001	0.8909	0.0074	<0.001
*Maximum limits (FAO/WHO)*	2	1	1	3	0.3	20	—	—

### 3.2 Essential oil yield

The hydrodistillation extraction of the flowering tops of *R. montana* yielded an average of 2.07% (v/w) of a yellowish essential oil with a strong aromatic odor (Table 4). This EO yield is higher than those reported in Algeria by Slougui et al. (2023) (0.67%). However, it is lower than those noted in Morocco by Barbouchi et al. (2021) (2.24% ± 0.02%) and in Algeria by Zeraib et al. (2021) (2.5%). Variations in essential oil yields arise from several factors, including the plant’s geographical location, climatic conditions, harvesting period, and the extraction method used. These differences in yield could be attributed to the specific characteristics of the plant material and the extraction conditions employed in each study.

**TABLE 4:** Essential Oil yield of *R. montana*.

*R. montana*	Yield (%)	Color	Odor
Flowering tops	2.07 ± 0.25	Yellow	Aromatic

### 3.3 Quality control of essential oil

Physicochemical properties such as acid value, peroxide value, and iodine value are among the key methods for verifying and controlling the quality of the essential oil extracted from the flowering tops of *R. montana*. These parameters are determined according to specific protocols. The results are presented in Table 5 and summarized below.

**TABLE 5:** Physicochemical properties of *R. montana* EO.

Properties	EO
Density (g/mL)	0.97 ± 0.01
Brix Degree	56.00 ± 0.05
Acid Value	1.12 ± 0.05
Iodine Value	13.00 ± 1.19
Peroxide Value	13.24 ± 0.98

The essential oil extracted from the studied plant recorded a density of 0.97. The AFNOR standard (2005) recommends a density between 0.906 and 0.990, suggesting that the essential oil extracted from the flowering tops of *R. montana* meets the standards for a very high-quality essential oil.

The acid value refers to the content of free fatty acids present in the EO. Indeed, fresh essential oils contain very little free acid (Fauconnier, 2006). Moreover, an essential oil is considered well-preserved if its acid value is less than 2. In this study, the EO extracted from the flowering tops of *R. montana* recorded an acid value of 1.12. Therefore, we conclude that the studied EO is well-preserved against any factors promoting degradation. Among the physicochemical parameters determined in this study is the iodine value, which measures the degree of unsaturation of the EO and directly affects its stability. In this work, the EO extracted from the flowering tops of *R. montana*. recorded an iodine value of 13 g/100 g of oil. As for the peroxide value, it is a crucial indicator of the quality and stability of the EO, reflecting its level of oxidation. The analyzed EO showed a peroxide value of 13.24 ± 0.98 mEq/kg. By comparing our results with the commercial standards, CODEX STAN 210-1999, we observe that the studied EO complies with the standards, allowing us to classify it as of good quality.

### 3.4 Chemical composition of the essential oil

The chromatogram of the essential oil (EO) of *R. montana* reveals a highly prominent main peak, clearly distinguishable from other less intense peaks (Supplementary Figure S1). This peak, detected at a retention time (RT) of 21.40 min, was identified as corresponding to the major compound 2-undecanone. Furthermore, the use of peak deconvolution enabled the effective separation of co-eluting compounds, thus ensuring a more accurate and detailed representation of the essential oil’s chemical profile.

**FIGURE 2:**
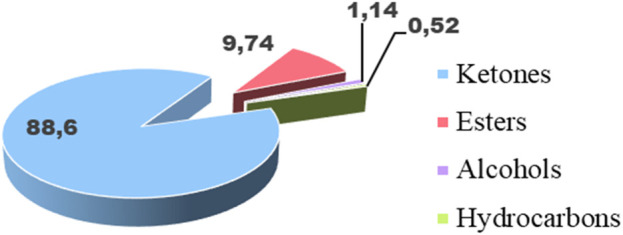
Distribution of the main chemical families of the EO of *R. montana*.

Based on chromatographic data and mass spectra, the chemical analysis identified 25 components, which together make up the entire composition of the essential oil (Table 6). The five most significant compounds, accounting for 95.55% of the total, are 2-undecanone (81.16%), decyl propanoate (9.33%), 2-dodecanone (1.99%), pulegone (1.86%), and 2-decanone (1.21%). It appears that the essential oil under study lacks monoterpenes (3.21%) and sesquiterpenes (0.49%), but contains other chemical classes (96.3%). These classes are primarily composed of aliphatic ketones (88.6%), followed by esters (9.74%). Meanwhile, alcohols (1.14%) and hydrocarbons (0.52%) are nearly absent (Figure 2).

**TABLE 6:** Chemical composition of the essential oil of *R. montana*.

N°	RT (min)	Compound	KI (Adams)	Area (%)	Mass	Formula
1	11.34	ο-Cymene	1026	0.03	134	C_10_H_14_
2	12.51	γ-Terpinene	1059	0.06	136	C_10_H_16_
3	14.08	2-Nonanone	1090	0.52	142	C_9_H_18_O
4	14.62	trans-Thujone	1114	0.92	152	C_10_H_16_O
5	16.08	iso-3-Thujanol	1138	0.04	152	C_10_H_16_O
6	16.22	Camphor	1146	0.04	152	C_10_H_16_O
7	17.28	Borneol	1169	0.07	154	C_10_H_18_O
8	17.38	Terpinen-4-ol	1177	0.05	154	C_10_H_20_O
9	17.67	2-Decanone	1192	1.21	156	C_10_H_16_O
10	19.47	Pulegone	1237	1.86	152	C_10_H_16_O
11	20.59	(5Z)-Octenol propanoate	1298	0.25	184	C_11_ H_20_ O_2_
12	21.31	2-Undecanone	1294	81.16	170	C_11_H_22_O
13	21.56	n-Undecanol	1370	0.80	172	C_11_H_24_O
14	22.08	Thymol	1290	0.03	150	C_10_H_14_O
15	22.31	Carvacrol	1299	0.11	150	C_10_H_14_O
16	23.40	2-Dodecanone	1389	1.99	184	C_12_H_34_O
17	24.70	Valeric acid,α-methylbenzyl ester	1400	0.04	206	C_13_H_18_O_2_
18	24.82	(E)-Caryophyllene	1419	0.24	204	C_15_H_24_
19	25.28	Decyl propanoate	1501	9.33	214	C_13_H_26_O_2_
20	26.68	Dauca-4(11),8-diene	1531	0.02	204	C_15_H_24_
21	27.05	α-trans-Bergamotene	1434	0.17	204	C_15_H_24_
22	27.30	Phenyl ethyl 2-methylbutanoate	1487	0.12	206	C_13_H_18_O_2_
23	27.42	2-Tridecanone	1496	0.88	198	C_13_H_26_O
24	31.74	Caryophylla-4(12),8(13)-dien-5β-ol	1640	0.04	220	C_15_H_24_O
25	36.67	6,10,14-Trimethylpentadecan-2-one	1800	0.02	268	C_18_H_36_O
		Hydrocarbon Monoterpenes				0.09
		Oxygenated Monoterpenes				3.12
		Hydrocarbon Sesquiterpenes				0.43
		Oxygenated Sesquiterpenes				0.06
		Others				96.3
		Total				100

The chemical composition described in this study shows similarities with the profile of *R. montana* from Morocco, particularly in terms of the dominance of 2-undecanone: 85.76% reported by Flouchi et al. (2024), and 63.97% by Benali et al. (2020b). In Algeria, comparative studies on the essential oils of *R. montana* collected from different regions have shown slight variations in the major components. Mohammedi et al. (2019), emphasized the impact of climatic conditions on the chemical composition of the essential oil, with its main components being 2-undecanone (27.2%–81.7%), 2-nonanone (1.9%–39.5%), and 2-nonanyl acetate (trace–24.8%). Zellagui et al. (2012) found that the major components of the first essential oil were 2-undecanone (60.1%), 2-nonanone (8.6%), monoethylhexyl phthalate (6.4%), and decanone (6.2%), while the second essential oil had 2-undecanone (90.4%), 2-nonanone (4%), and decanone (1.4%).

It is equally important to highlight the influence of the developmental stage and the condition of the plants (whether fresh or dried) on the composition of EO. In this context, the fresh aerial parts collected in eastern Algeria are primarily composed of undecan-2-one (37.74%), resorcinol (27.66%), and 2-acetoxytetradecane (9.19%) (Djarri et al., 2013), whereas these compounds were not identified in other Algerian samples. In Tunisia, Hammami et al. (2015) found that the EO extracted from dried leaves was predominantly composed of 1-butene (38.33%), methylcyclopropane (15.47%), 2-butene (22.56%), and caryophyllene oxide (8.18%). Conversely, in the same country, Yosra et al. (2019) reported a chemical profile of EO extracted from dried leaves that was dominated by the same major compounds found in Moroccan and Algerian samples, particularly 2-undecanone (86.77%), followed by 2-decanone (4.91%) and 2-nonanone (23.62%).

It is important to highlight that the abundance of 2-undecanone in the EO of *Ruta* species has made it a relevant chemotaxonomic marker for this genus (Bennaoum et al., 2017). Thus, 2-undecanone appears to be an indispensable compound in the EO of *R. montana*, given its presence in all the EOs of the species from Morocco, Algeria, and Tunisia, regardless of the region or the plant’s growth conditions. The variation in cultivation environments is mainly reflected in the differing percentages of this compound depending on the regions.

### 3.5 Phytochemical screening

The results of the phytochemical screening on the plant powder of the flowering tops of *R. montana* are illustrated in Table 7.

**TABLE 7:** Results of phytochemical tests conducted on extracts of *R. montana*.

Chemical families		*R. montana*
Polysaccharides		++
Reducing Sugars		++
Lipids		+++
Proteins	Biuret reaction	+++
	Xanthoproteic reaction	+
Tannins	Catechic	++
	Gallic	++
Flavonoids	Free Flavonoids	++ (flavones)
	Anthocyanins	++
	Leucoanthocyanins	+
Alkaloids	Dragendorff	++
	Mayer	++
Sterols and Triterpenes		++
Saponosides		++
Mucilages		−

Classification: (−): Absence of the desired compound in the reaction; +: Low presence of the tested compound; ++: Presence of the compound, with relatively high concentration; +++: Presence of the compound with high concentration.

This qualitative analysis revealed the diversity of primary and secondary metabolites in *R. montana*. The plant is particularly rich in lipids and proteins, and moderately rich in reducing sugars and glycogens. Regarding secondary metabolites, the various extracts showed a moderate presence of alkaloids, flavones, and both gallic and condensed tannins. A positive reaction was also observed for saponosides, sterols, and triterpenes, whereas mucilages showed a negative reaction. The richness of this plant’s extracts in secondary metabolites explains its use in traditional medicine to treat numerous diseases. The results of this study align with those of Drioiche et al. (2020a), who found that the aerial parts of Moroccan *R. montana L.* contained alkaloids, mucilages, triterpenes, sterols, flavonoids, leucoanthocyanins, and tannins. Similarly, Drioueche et al. (2021) discovered that the polar extract of the aerial parts of mountain rue from Algeria contained alkaloids, flavonoids, tannins, and coumarins.

### 3.6 HPLC/UV ESI-MS analysis of *R. montana* extracts

**FIGURE 3:**
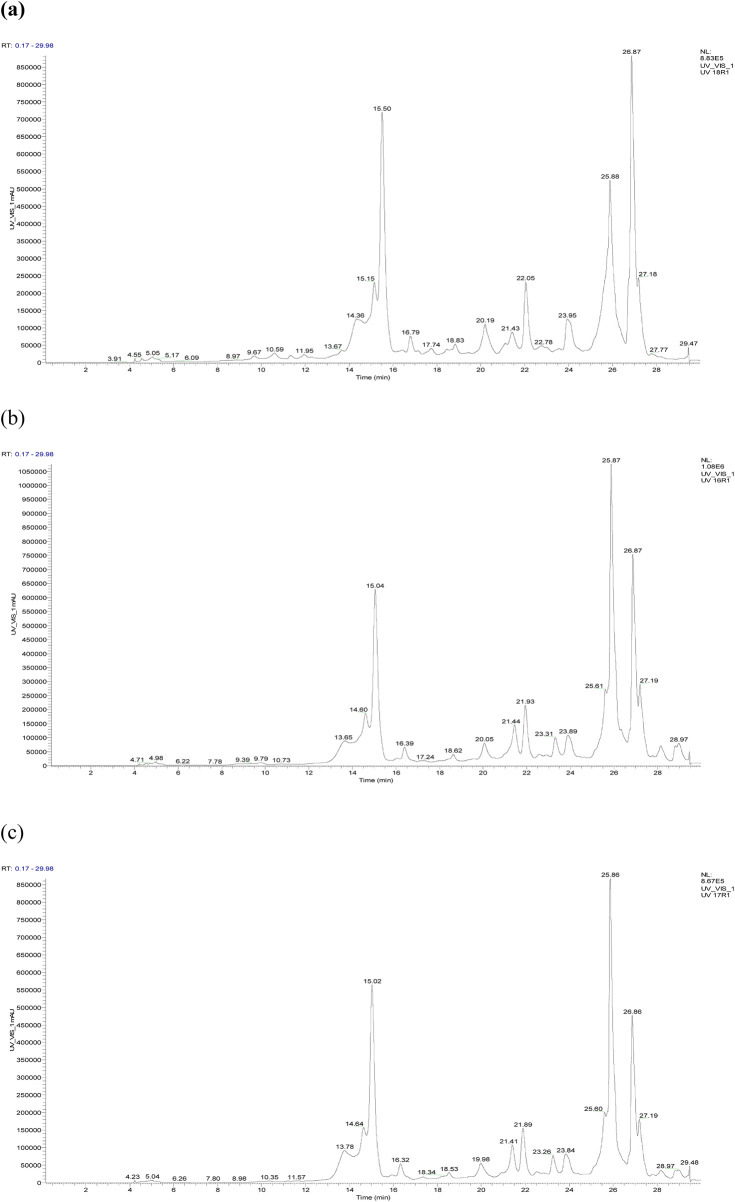
HPLC chromatogram of *R. montana* compounds of aqueous extract **(a)**, hydroethanolic extract **(b)**, and hydromethanolic extract **(c)**.

The chromatographic profile, shown in Figure 3, displays the peaks of phenolic and non-phenolic compounds derived from the decoction and hydroethanolic and hydromethanolic extracts of the flowering tops of *R. montana*, along with their retention times and relative abundances. The HPLC/UV-ESI-MS technique was used to evaluate the decoction, hydromethanolic, and hydroethanolic extracts of *R. montana*. By analyzing the mass spectra in parallel with the chromatogram, a total of 35 molecules were identified and are listed in Table 8. The chromatographic profile highlights the diverse richness of the analyzed extracts in various molecules.

**TABLE 8:** List of compounds identified in the extracts of the flowering tops of *R. montana* by HPLC/UV-ESI-MS.

N°	RT (min)	Molecules	Classes	Nominal masses	[M-H]^−^ (m/z)	Fragment ions (m/z)	E (1)	E (2)	E (3)
1	4,23	2,5-Dihydro-2-furancarboxylic acid	Others	114	113	113-97-69	0.11	0.05	0.1
2	5,05	Salicylic acid	Phenolic compound	138	137	139-277	0.23	0.03	0.1
3	8,97	Caffeic acid	Phenolic acid	180	179	135-107	0	0	0.35
4	9,67	Ferulic acid derivative	Phenolic acid	222	221	149-117	0.25	0.36	0.73
8	9,8	Luteolin	Flavonoid	286	285	175-151-133	0	0.17	0
5	10,59	Vanillic acid	Phenolic acid	168	167	167-123	0.1	0.06	0.98
6	11,34	Formononetin	Flavonoid	268	267	251-223	0	0	0.41
6	11,57	Propyl gallate	Phenolic compound	212	211	211-137-107	0.84	0.03	0
7	11,95	Umbelliferone	Coumarin	162	161	161-133	0.65	0.1	0.55
8	12,27	Protocatechuic acid	Phenolic acid	154	153	153-109	0.11	0.01	0.35
9	13,65	Gallic acid	Phenolic acid	170	169	125-107	0.23	0.13	1.37
10	14,36	Embelin	Phenolic compound	294	293	293-184	6.25	4.01	6.12
11	14,6	Ferulic acid	Phenolic acid	194	193	193-175-147	6.1	8.44	6.49
12	15,5	p-Coumaroylquinic acid	Phenolic acid	338	337	337-435	15.5	18.4	15.08
13	16,43	Harpagide	Others (Iridoid)	364	363	363-195-179	0	0	0.98
14	16,79	3-Feruloylquinic acid	Phenolic acid	368	367	367-191-173	1.72	1.08	1.46
15	17,13	Chrysin	Flavonoid	254	253	253-143-107	0	0	0.54
13	17,34	Methyl rosmarinate	Phenolic compound	374	373	373-179-135	0.69	0	0
16	17,74	1-Caffeoyl-beta-D-glucose	Phenolic compound	342	341	341-177-135	0.81	0.7	1.3
14	18,34	Amentoflavone	Flavonoid	538	537	355-179-151	0.74	0.35	0
17	18,45	Oleuroside	Phenolic compound	540	539	539-301-123	0	0	0.9
18	18,83	Stigmasterol	Others (Steroid)	412	411	411-227-119	0	0	1.42
16	19,22	Vanillic acid glucoside	Phenolic compound	330	329	329-169-151	0.42	0	0
19	19,44	Quercetin 3-rutinoside-7-rhamnoside	Flavonoid	756	755	755-467-301-179	3.44	3.5	0.66
17	19,51	Catechin-4-ol 3-O-β-D-galactopyranoside	Flavonoid	468	467	467-303-171	0.4	0	0
20	20,19	Valoneic acid dilactone	Phenolic compound	470	469	469-307-179	2.77	1.82	3.88
20	20,93	Quercetin 3-(6″-malonyl-glucoside)	Flavonoid	550	549	549-505	0.73	0	0
21	21,12	Alyssonoside	Flavonoid	770	769	769-463	0	0	1.18
22	21,43	Rutin	Flavonoid	610	609	609-301-255	4.09	4.31	2.37
23	22,05	Chlorogenic Acid	Phenolic acid	354	353	707 [2M−H]−, 191, 179, 135	1.32	0.51	4.75
24	22,78	Quercetin-3-O-pentosyl-pentoside	Flavonoid	566	565	565-303-151	0.23	0	1.24
24	22,81	Quercetin-3′-glucoside	Flavonoid	464	463	463-447-301	0.74	0	0
24	22,89	Myricetin glucuronide	Flavonoid	494	493	493-317	0	0.1	0
25	22,98	Gallic acid 4-O-(6-galloylglucoside)	Phenolic acid	484	483	483-331-169	0	0.13	0.77
25	23,26	Oleuropeic acid 8-O-glucoside	Phenolic compound	346	345	345-171-143	2.14	0	0
25	23,31	Betonyoside A	Phenolic compound	654	653	653-491-271	0	1.52	0
26	23,55	Rhamnetin 3-rutinoside	Flavonoid	624	623	632-301-169	3.31	2.62	0.97
27	23,95	3,4-Di-O-galloylquinic acid	Phenolic acid	496	495	495-341-169	0	0.21	4.19
27	24,36	Cyanidin 3-arabinoside	Flavonoid	454	453	453-303-179	0.5	0	0
29	25,6	Rhamnetin 3-rhamnoside	Flavonoid	462	461	461-315-151	6.27	3.75	0.27
30	25,87	Rosmarinic acid 3′-glucoside	Phenolic compound	522	521	521-365-197	21.9	23.8	20.03
31	26,87	Quercitrin	Flavonoid	448	447	447-285-151	10.46	15.5	15.42
32	27,18	Salvigenin	Flavonoid	328	327	327-297-167	0	0	3.89
31	27,19	(+)-Gallocatechin	Flavonoid	306	305	305-275-125	0	5.3	0
33	27,41	Taxifolin	Flavonoid	304	303	303-273-125	4.82	1.33	0.25
32	27,77	7-Methoxyflavone	Flavonoid	252	251	251-221-181	1.29	0.33	0
34	29,54	Syringetin-3-O-hexoside	Flavonoid	508	507	507-345	0	1.07	0.11
35	30,55	Caffeoyl-feruloyltartaric acid	Phenolic acid	488	487	487-457-375	0.71	0.23	0.15

**FIGURE 4:**
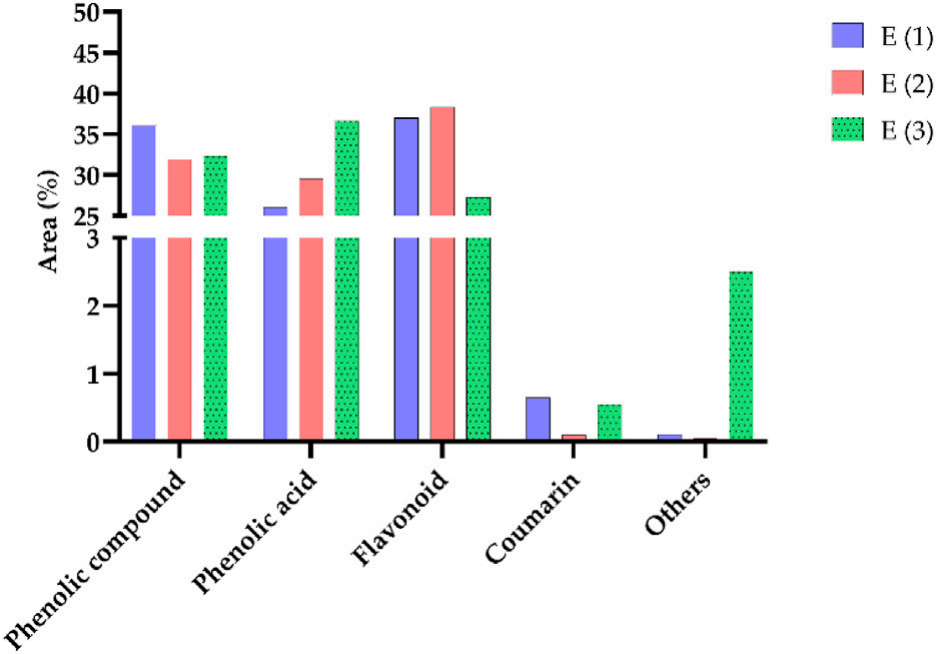
Distribution of different classes of compounds in the analyzed samples.

The distribution of chemical classes in the extracts of *R. montana*, as presented in Figure 4, reveals that phenolic compounds predominate across all extracts, with the highest proportion observed in the hydromethanolic extract (36.05%), followed by the decoction (32.33%) and the hydroethanolic extract (31.91%). Phenolic acids are most abundant in the decoction (36.67%), demonstrating the efficiency of water as a solvent for their extraction, whereas flavonoids are better extracted using mixed solvents such as hydromethanolic and hydroethanolic solutions (37.02% and 38.33%, respectively). Coumarins, although present in smaller quantities, are slightly more concentrated in the hydromethanolic extract (0.65%), while compounds classified as “others” are primarily found in the decoction (2.50%). These findings highlight the influence of solvents on the extraction process, with mixed solvents favoring flavonoids and water being more suitable for phenolic acids. From a biological perspective, the phenolic acids and flavonoids, owing to their antioxidant and anti-inflammatory properties, offer promising prospects for therapeutic and nutraceutical applications.

**FIGURE 5:**
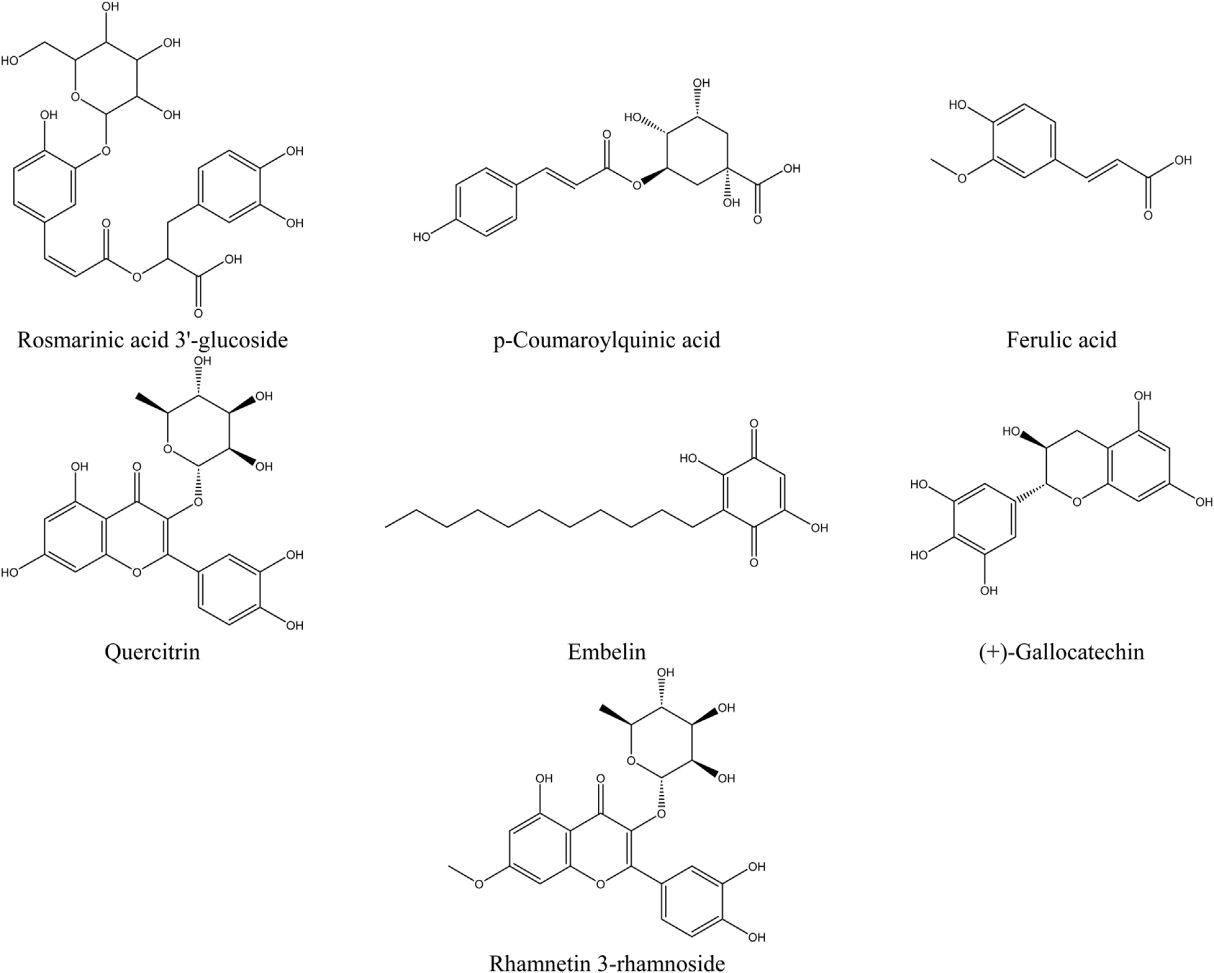
Structure of the main compounds identified in the extracts of the flowering tops of *R. montana*.

The primary compounds identified in the extracts include rosmarinic acid 3′-glucoside, which is present in significant quantities in all extracts, reaching up to 23.8% in the hydroethanolic extract. p-Coumaroylquinic acid is also highly abundant, with a concentration of 18.4% in the hydroethanolic extract. Ferulic acid stands out due to its dominance in the hydroethanolic extract, where it reaches 8.44%. Embelin is notably present in all extracts, with a maximum concentration of 6.25%. Quercitrin, a major flavonoid, accounts for up to 15.5% in these extracts. Additionally, we recorded a content of Rhamnetin 3-rhamnoside in the hydromethanolic extract with a concentration of 6.27%. Furthermore, certain specific compounds, such as (+)-Gallocatechin, are detected exclusively in the hydroethanolic extract. Figure 5, which presents the chemical structures of the main identified compounds, complements this analysis by providing precise structural details.

The analysis of compounds by mass spectrometry in negative ion mode (ESI-MS) allows for the exploration of their specific fragmentation patterns, thereby providing valuable insights into their molecular structure. The ESI-MS spectrum of Rosmarinic acid 3′-glucoside displays a molecular ion [M−H]^−^ at m/z 521, fragmenting through the loss of a glucose residue (162 u) to m/z 365, followed by the loss of a rosmarinic acid fragment to reach m/z 197. The p-Coumaroylquinic acid, with a [M−H]^−^ ion at m/z 337, suggests the loss of coumaroyl or quinic acid fragments. The fragmentation of Ferulic acid generates fragments at m/z 175 and 147, corresponding to the loss of a water molecule (18 u) and a methoxy group (28 u), respectively. Quercitrin exhibits a [M−H]^−^ ion at m/z 447, with a typical flavonoid fragmentation involving the loss of a rhamnose residue (162 u) to form the aglycone quercetin (m/z 285), followed by an aromatic cleavage at m/z 151. Regarding Embelin, the [M−H]^−^ ion at m/z 293 loses a bulky fragment (109 u) to reach m/z 184. (+)-Gallocatechin fragments into m/z 275 (loss of 30 u, corresponding to a water molecule), then into m/z 125 through cleavage of the flavonoid ring. Finally, Rhamnetin 3-rhamnoside presents a [M−H]^−^ ion at m/z 461, fragmenting through the loss of a rhamnose residue (146 u) to form the aglycone rhamnetin (m/z 315), followed by an aromatic cleavage at m/z 151. These results highlight common fragmentation mechanisms, including the loss of carbohydrate residues, specific functional groups, and cleavages at aromatic rings or side chains, enabling precise characterization of the studied molecules.

In the literature, the phenolic profile of *R. montana* has been scarcely studied. Khadhri et al. (2017) reported the abundant presence of flavonoid-type polyphenols in the decoction, as well as phenolic acids in the decoction of *R. montana* originating from Tunisia. Szewczyk et al. (2023) noted the existence of catechin in the methanolic extract of *R. montana* L. cultivated *in vitro*. Toker and collaborators (Toker et al., 1998) determined the rutin content in the methanolic extract of the aerial parts of *R. montana* L. from Turkey, using the HPLC method, to be 6.79 µg/mL.

Moreover, a study conducted by Pacifico et al. (2016) demonstrated that the hydroalcoholic extract of *R. graveolens* leaves collected in Italy contains rutin, isorhamnetin-3-O-rutinoside, 4-O-p-coumaroylquinic acid, and 4-O-feruloylquinic acid. Additionally, this study highlighted the variation in the chemical composition of this species depending on the harvest season.

All the major compounds quantified in the three extracts of *R. montana* possess therapeutic properties. For example, rosmarinic acid glucoside exhibits significant anticancer, anti-angiogenic, antioxidant, anti-inflammatory, and antimicrobial properties (Budzianowski et al., 2023). In turn, p-coumaroylquinic acid is distinguished by its numerous biological activities, including antioxidant, anticancer, antimicrobial, antiviral, anti-inflammatory, antiplatelet aggregation, anxiolytic, antipyretic, analgesic, and antiarthritic effects (Pei et al., 2016). Additionally, quercitrin demonstrates potential antioxidant, anti-inflammatory, antiviral, and anticancer properties, as well as the ability to alleviate certain cardiovascular diseases (Ulusoy and Sanlier, 2020). Ferulic acid is also recognized for its antioxidant, anticancer, anti-inflammatory, hepatoprotective, antimicrobial, and antiviral effects (Pyrzynska, 2024). Similarly, rhamnetin possesses various pharmacological properties, including antioxidant, anticancer, anti-inflammatory, antiviral, and antibacterial activities (Medeiros et al., 2022). Finally, embelin exhibits antimicrobial, antioxidant, analgesic, anti-inflammatory, anxiolytic, antifertility, antimalarial, and anticancer properties (Basha et al., 2022).

### 3.7 Extraction yield, total polyphenol, flavonoid, and catechic tannin content

**FIGURE 6:**
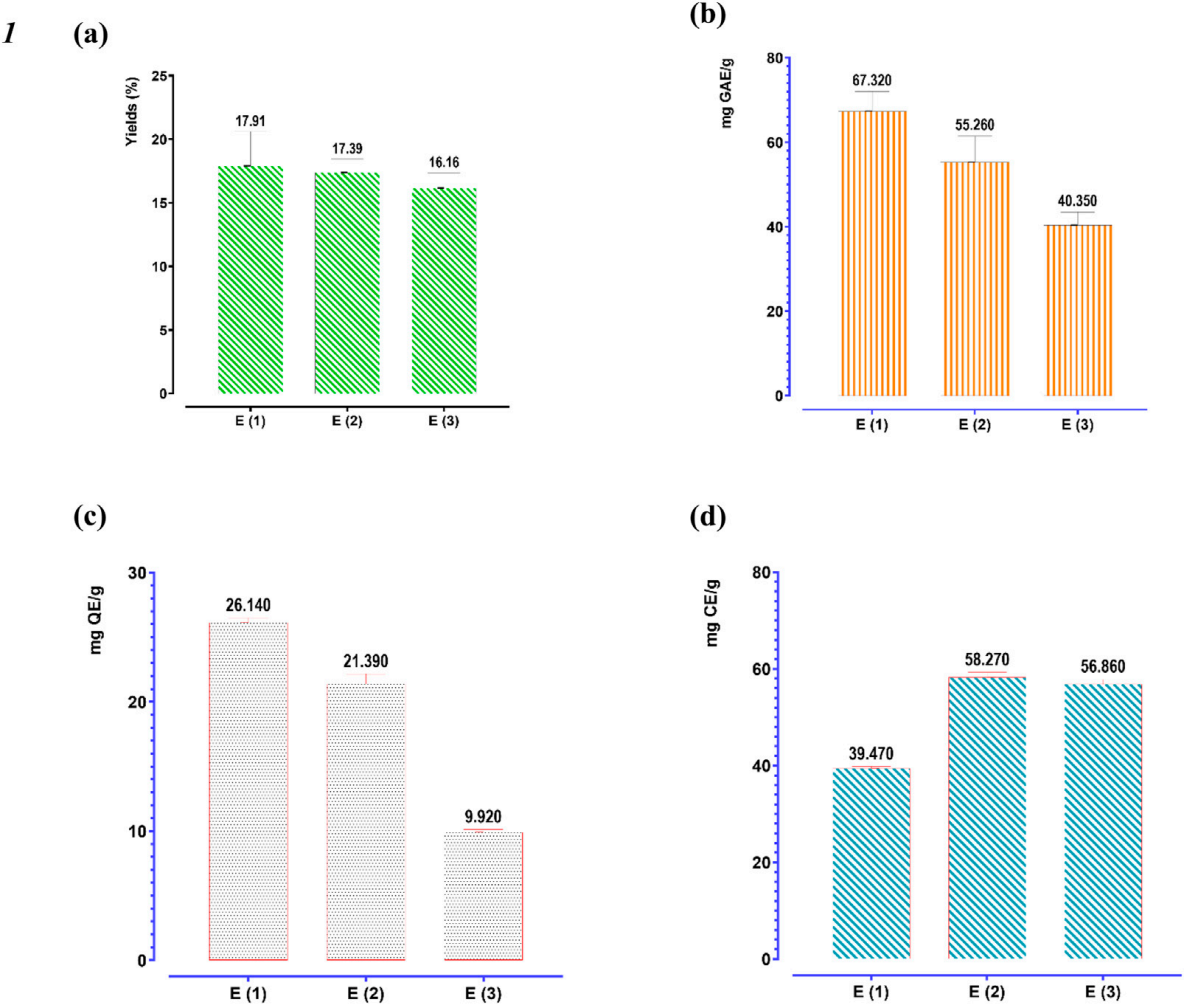
Extraction yield **(a)**, phenolic content **(b)**, flavonoid **(c)**, and condensed tannins contents **(d)** of three extracts of *R. montana.* The means display a significant difference (*p < 0.001*).

Figure 6 presents the average extraction yields, as well as the polyphenol, flavonoid, and catechin tannin contents of *R. montana*, based on the extraction methods employed.

The extraction yields depend on both the solvent and the extraction method. We observe that aqueous methanol yielded the highest extraction yield (17.91%), followed by aqueous ethanol (17.39%). In contrast, decoction recorded the lowest extraction yield (16.16%). Regarding the quantification of polyphenols, flavonoids, and condensed tannins, their contents are expressed in mg GAE/g of extract, mg QE/g of extract, and mg CE/g of extract, respectively. It appears that the flowering tops of R. montana L. are rich in these molecules. Indeed, aqueous methanol is the most effective extractor of polyphenols and flavonoids for R. montana L., with average values of 67.32 mg GAE/g of extract and 26.14 mg QE/g of extract, respectively. This is followed by aqueous ethanol, which shows contents of approximately 55.26 mg GAE/g of extract and 21.39 mg QE/g of extract, respectively. However, the decoction recorded the lowest polyphenol and flavonoid contents, amounting to 40.35 mg GAE/g of extract and 9.92 mg QE/g of extract, respectively.

Unlike polyphenols and flavonoids, the condensed tannins of *R. montana* were more soluble in aqueous ethanol (58.27 mg CE/g of extract) than in the decoction (58.27 mg CE/g of extract) and aqueous methanol (39.47 mg CE/g of extract).

The contents of Mountain Rue extracts found in this study are significantly higher than those reported by Szewczyk et al. (2023), where the polyphenol and condensed tannin contents of the methanolic extract prepared from *in vitro* cultivated biomass were 41.61 mg GAE/g of extract and 10.97 mg CE/g of extract, respectively. Similarly, Drioiche et al. (2020a) reported that the polyphenol and flavonoid contents of the hydro-methanolic extract from the aerial parts of Moroccan R. montana L. were 33.684 mg GAE/g of extract and 0.843 mg QE/g of extract, respectively. Merghem and Dahamna (2020) noted that the ethyl acetate extract of R. montana L. from Algeria contained the highest quantities of polyphenols, tannins, and flavonoids, with values of 257.1 µg gallic acid equivalent/mg of extract, 251 tannic acid equivalent/mg of extract, 117.4 µg quercetin equivalent/mg of extract, and 139.5 µg rutin equivalent/mg of extract, respectively. Our results are lower than those reported by Benali et al. (2020a), where the highest phenolic compound and flavonoid contents were found in the methanolic extract of *R. montana* collected from northeastern Morocco, with values of 117.70 mg GAE/g of extract and 77.00 mg RE/g of extract.

The variation in polyphenol and flavonoid levels among the analyzed extracts can be explained by the difference in the solubility of phenolic compounds, which is influenced by the extraction temperature as well as the structure of these compounds. Additionally, the choice of extraction method can also have a significant impact on the extraction and quantification of polyphenols, flavonoids, and condensed tannins (Alara et al., 2021; Lama-Muñoz and Contreras, 2022; El Mannoubi, 2023).

### 3.8 Antioxidant activity of the EO and phenolic extracts

#### 3.8.1 Antioxidant activity of the EO

**FIGURE 7:**
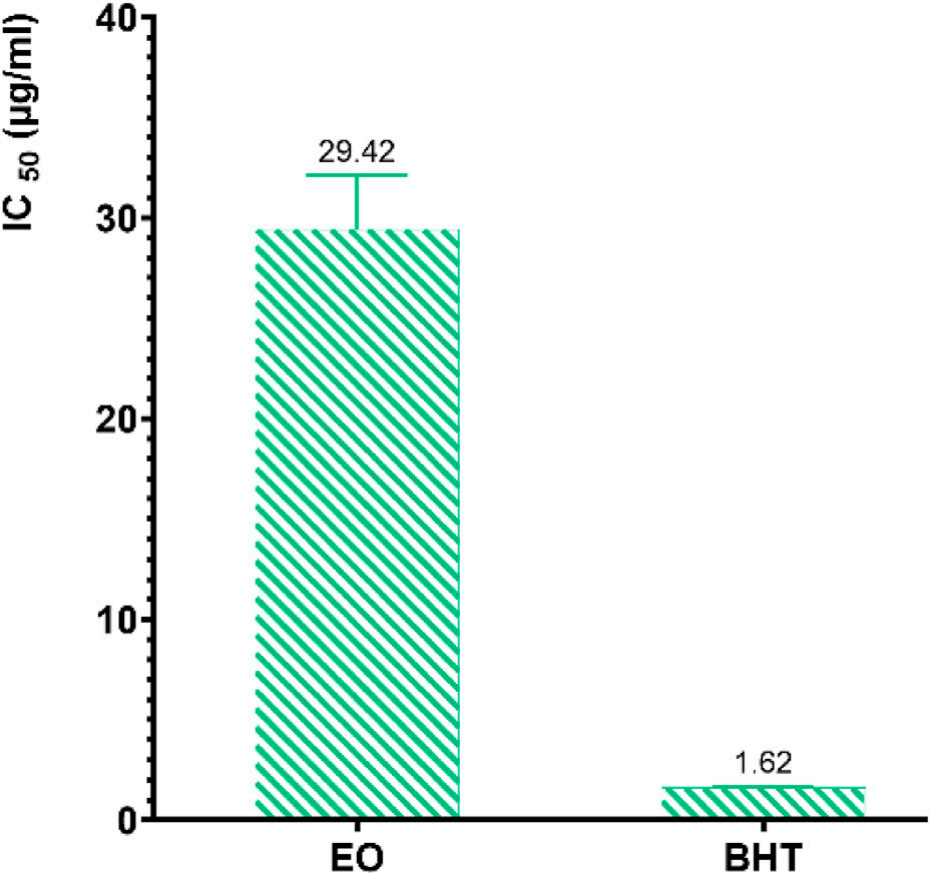
IC_50_ values of *R. montana* and the standard antioxidant BHT using the DPPH method.

The results reveal that the inhibition of DPPH radicals varies depending on the concentration of the EO from *R. montana.* According to the IC_50_ values presented in Figure 7, the standard antioxidant butylated hydroxytoluene (BHT) exhibits a significantly greater antioxidant capacity compared to the EO of *R. montana.* Indeed, the latter shows a weak ability to reduce DPPH free radicals, with an IC_50_ value 18.16 times higher than that of BHT.

The antioxidant potential of the EO of *R. montana* has already been examined by various researchers. In Morocco, Benali et al. (2020b) indicated that the EO from the aerial parts of mountain rue collected in Taza, Morocco, which contains components such as 2-undecanone (63.97%), camphor (3.82%), and cyclopropanecarboxylic acid (3.66%), exhibits a remarkable ability to reduce free radicals (IC_50_ = 244.62 µg/mL). Meanwhile, the reducing power of this EO, expressed in terms of micrograms of ascorbic acid equivalent per gram of EO, was 1.39 µg/g. In the same country, Drioiche et al. (2020a) found through the DPPH method that the EO of the aerial parts of *R. montana* collected in Boulemane, Morocco, with 2-undecanone (82.62%), 2-undecanol (2.87%), and 2-undecanol acetate (2.13%) as its main compounds, has an IC50 (548.5 μg/mL) 10.28 times higher than that of ascorbic acid (53.35 μg/mL). In Algeria, Mohammedi et al. (2019) confirmed through the DPPH method the significant antioxidant capacity of all EOs isolated from *R. montana* collected in seven different regions. In particular, EO samples 6 and 7 exhibited strong DPPH inhibition activity with IC_50_ values of 49.6 and 50.2 µg/L, respectively. Moreover, these values were not influenced by the cultivation site. These researchers attributed the remarkable antioxidant activity of EOs 6 and 7 to their high content of 2-undecanone, which was 81.7% and 78.6%, respectively (Mohammedi et al., 2019). However, the study by Slougui et al. (2023) reported the inactivity of the EO extracted from the aerial parts of Algerian *R. montana* using the DPPH, CUPRAC, ABTS, and FRAP methods, with its main compounds being 2-nonanone (24.93%) and 2-undecanone (22.62%).

The antioxidant activity of EOs generally arises from the complex interaction between the various chemical components present in the EOs, which can act either antagonistically or synergistically. It should also be noted that EOs are composed of a complicated mixture of different classes of compounds, including aliphatic alcohols, monoterpenes, sesquiterpenes, ketones, aldehydes, and acids. For this reason, it is often difficult to fully understand the interactions and identify the compounds responsible for the antioxidant activity.

In the literature, the EOs of the *Ruta* genus have demonstrated promising antioxidant potential through various mechanisms, depending on the concentration. Moreover, the EOs of *Ruta* species are generally characterized by a mixture of ketones, which can represent up to 84% of the EO (Bertrand et al., 2003; Kambouche et al., 2008). However, significant disparities exist between species, both in terms of the concentration of these compounds and the presence of other compounds in high concentrations. This can be attributed to the fact that the composition of EOs isolated from *Ruta* species is strongly influenced by a combination of intrinsic and extrinsic factors, such as genetic and environmental factors. In this context, a study by Jaradat et al. (2017) highlighted the influence of the collection region of *R. chalepensis* leaves on the antioxidant activity of its EO. These researchers found using the DPPH method that the EO of *R. chalepensis* from Jerusalem exhibited the highest antioxidant power (IC_50_ = 6.9 µg/mL), followed by those from Hebron (IC_50_ = 7.8 µg/mL) and Jenin (IC_50_ = 19.9 µg/mL). Another study revealed the influence of vegetative stages, flowering, and different organs of *R. chalepensis* on the chelating activity of its EOs. EOs extracted from the stems and flowers of *R. chalepensis* harvested during the flowering stage exhibited higher chelating power than those from the leaves (Krayni et al., 2015). Recently, a study evaluated the antioxidant power of the EO extracted from the aerial parts of Saudi *R. chalepensis* using three *in vitro* methods. The authors observed powerful concentration-dependent antioxidant activity using DPPH, nitric oxide scavenging, and ferric ion reducing power (FRAP) methods, with IC_50_ values of 40.26%, 36.88%, and 19.09%, respectively (Aati et al., 2023). The EO extracted from the aerial parts of *R. graveolens* from Romania, containing 2-undecanone (76.19%) and 2-nonanone (7.83%), was capable of reducing the stable DPPH free radical with an IC_50_ value of 0.25 mg/mL (Jianu et al., 2021). In summary, these studies highlight the antioxidant effects of EOs from *Ruta* species, indicating their potential for commercial use as natural antioxidants.

#### 3.8.2 Antioxidant activity of extracts

The antioxidant activity of the decoction and the hydromethanolic and hydroethanolic extracts of *R. montana* was determined using three methods: DPPH radical scavenging, ferric ion reducing power, and total antioxidant capacity. By varying the concentrations of the extracts (mg/mL), we were able to determine the IC_50_ (the concentration of extract required to reduce 50% of the DPPH free radical levels) and the EC_50_ (the effective concentration at which the absorbance equals 0.5) for the FRAP method.

**FIGURE 8:**
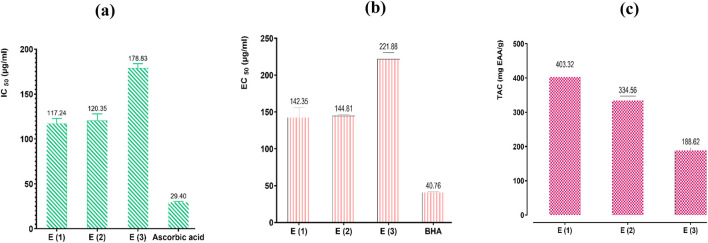
Antioxidant effects of extracts of *R. montana* and standards (BHA and ascorbic acid) by DPPH **(a)**, FRAP **(b)**, and TAC **(c)** methods. Values are expressed as mean ± SEM (n = 3). The means display a significant difference (*p < 0.001*).

According to the results presented in Figure 8, the IC_50_ and EC_50_ values for ascorbic acid and the EC_50_ of BHA, used as reference molecules in the DPPH and FRAP methods, are significantly lower than those of the extracts, indicating a high antioxidant activity of the standards.

The estimation of the antioxidant capacity of the three extracts showed considerable variability depending on the nature of the solvent used for extraction. Indeed, the hydromethanolic extract, which exhibited high levels of polyphenols and flavonoids, stands out from the other extracts through the three methods with significantly higher radical scavenging power, ferric ion reducing power, and Mo(VI) molybdate reducing power. Its strong antioxidant activity is reflected in a total antioxidant capacity of 403.32 mg EAA/g of extract, with IC_50_ values of 117.24 and 142.35 µg/mL for the DPPH and FRAP methods, respectively. It is followed by the hydroethanolic extract, which displays moderate antioxidant power with a total antioxidant capacity of 334.56 mg EAA/g of extract, with IC_50_ and EC_50_ values of 120.35 and 144.81 µg/mL for the DPPH and FRAP methods, respectively. The decoction, on the other hand, showed the weakest antioxidant powers, with a total antioxidant capacity of 188.62 mg EAA/g of extract, with IC_50_ and EC_50_ values of 178.83 µg/mL and 221.88 µg/mL for the DPPH and FRAP methods, respectively.

The difference in antioxidant activity observed between the *R. montana* extracts is specifically attributed to the levels of polyphenols, flavonoids, and other aromatic compounds present. In our study, referring to the results of phenolic compound assays, we found that the three extracts analyzed contain high levels of phenolic compounds, with the hydromethanolic extract recording the highest content of polyphenols and flavonoids. This is followed by the hydroethanolic extract and the decoction, which is frequently used in traditional medicine.

Furthermore, the results of chromatographic analyses performed using HPLC-MS on the decoction and the hydroethanolic and hydromethanolic extracts from the flowering tops of *R. montana*. revealed great diversity in their chemical profiles. This diversity is clearly illustrated by the presence of several chemical families such as phenolic acids, flavonoids, and benzoquinones. In the hydromethanolic extract, the main constituent is rosmarinic acid glucoside (21.9%), followed by p-coumaroylquinic acid (15.5%), quercitrin (15.5%), rhamnetin 3-rhamnoside (6.27%), embelin (6.25%), and ferulic acid (6.1%). In the hydroethanolic extract, the dominant compound is rosmarinic acid glucoside (23.8%), followed by p-coumaroylquinic acid (18.4%), quercitrin (15.5%), ferulic acid (8.44%), and gallocatechin (5.3%). In the decoction, the major component is rhamnetin 3-rhamnoside (20.03%), followed by rosmarinic acid glucoside (15.42%), quercitrin (15.42%), p-coumaroylquinic acid (15.08%), 4-di-O-galloylquinic acid (8.94%), ferulic acid (6.49%), and embelin (6.12%).

Similar results are reported by Benali et al. (2020a), where the methanolic extract of *R. montana* L. collected from Taza (Northeast Morocco) exhibited superior antioxidant activity compared to the ethanolic extract. These researchers found, using the DPPH and Reducing power methods (TAC), that the IC_50_ values for the methanolic extract were 10.66 µg/mL and 66.66 mg AAE/g of extract, respectively, while for the ethanolic extract, the IC_50_ values were 13.00 ± 1.73 µg/mL and 36.33 mg AAE/g of extract, respectively. Merghem and Dahamna (2020) confirmed the antioxidant potential of the aerial parts of *R. montana* using the DPPH free radical scavenging and ferrous ion chelation tests. In this study, the aqueous extract (IC_50_ = 83 µg/mL) and the methanolic extract (IC_50_ = 67 µg/mL) exhibited remarkable radical scavenging power, surpassing those of quercetin (IC_50_ = 3.491 mg/mL) and rutin (IC_50_ = 4.179 mg/mL), which were used as reference antioxidants.

Also, through the ferrous ion chelation test, the methanolic extract (IC_50_ = 21 µg/mL) and aqueous extract (IC_50_ = 5 µg/mL) exhibited significant activity. Slougui et al. (2023) reported the antioxidant effect of the ethyl acetate extract of the aerial parts of *R. montana* L. from Algeria using the FRAP and DPPH methods, noting IC_50_ values of 84.01 and 135.40 μg/mL, respectively. Another study found, using the DPPH method, that the antioxidant potential of the hydromethanolic extract obtained by Soxhlet from the aerial parts of *R. montana*. from Morocco was superior to that of the methanolic macerate, with IC_50_ values of 1097.2 μg/mL and 1332.8 μg/mL, respectively (Drioiche et al., 2020a).

### 3.9 Correlation between phenolic content and antioxidant activities of *R. montana* extracts

The results obtained previously demonstrate the relationship between phenolic compounds and the antioxidant activity of the three *R. montana* extracts. This is illustrated in this context (Table 9).

**TABLE 9:** Linear correlation coefficients (r) between polyphenol content (PC), flavonoid content (FC), condensed tannin content (CT), and the antioxidant activity of *R. montana* extracts.

	PC	FC	CT	DPPH	FRAP	TCA
PC	1	0.985	−0.795	0.923	0.913	0.990
FC	0.985	1	−0.678	0.976	0.970	0.9995
CT	−0.795	−0.678	1	−0.500	−0.478	−0.700
DPPH	0.923	0.976	−0.500	1	0.9997	0.969
FRAP	0.913	0.970	−0.478	0.9997	1	0.962
TCA	0.990	0.9995	−0.700	0.969	0.962	1

The linear correlation coefficients range from −0.700 to 0.9997. The antioxidant capacity of the extracts appears to be influenced primarily by the total polyphenol and flavonoid contents. These compounds showed high coefficients in comparison to condensed tannins (0.913 ≤ r ≤ 0.9995). In contrast, a negative correlation was observed between condensed tannins and the three antioxidant activities, namely the DPPH method (r = −0.5), the FRAP method (r = −0.478), and total antioxidant capacity (r = −0.7). This result leads us to suggest that condensed tannins do not influence the antioxidant activity of *R. montana* L. extracts. Similar results have linked the group of condensed tannins to phenolic compounds (r = −0.795) and flavonoids (r = −0.678).

On the other hand, we observe a very strong correlation between the IC_50_ and EC_50_ values from the DPPH and FRAP tests (r = 0.9997), between the IC_50_ values of DPPH and TCA (r = 0.969), and between the EC_50_ values of FRAP and TCA (r = 0.962). This indicates that the antioxidant activities demonstrated by these three tests are likely provided by the same active molecules.

### 3.10 Antimicrobial activity of *R. montana* extracts and essential oil

Tables 10, 11 present the minimum inhibitory concentrations (MIC), minimum fungicidal concentrations (MFC), and minimum bactericidal concentrations (MBC) expressed in mg/mL for the extracts and EO of *R. montana* against the tested microorganisms.

**TABLE 10:** MIC and MBC (mg/mL) of the extracts of *R. montana*, and MIC (µg/mL) of antibiotics against the microbial strains studied.

Bacterial strains	E (1)			E (2)			E (3)			EO			Antibiotics *			
	CMI	CMB	CMB/CMI	CMI	CMB	CMB/CMI	CMI	CMB	CMB/CMI	CMI	CMB	CMB/CMI	Gentamycin	Amoxicillin–Clavulanate	Vancomycin	Trimethoprim-Sulfamethoxazole
*E. cloacae*	25	25	1	25	25	1	50	50	1	50	50	1	>4	>8/2		>4/76
*K.pneumoniae*	>100	>100	—	50	50	1	>100	>100	—	50	50	1	≤1	≤2/2		≤1/19
*E. coli*	>100	>100	—	>100	>100	—	>100	>100	—	50	50	1	2	8/2		≤1/19
*S. aureus*	25	25	1	25	25	1	>100	>100	—	100	100	1	<0.5		2	<10
*S. epidermidis*	25	50	2	50	50	1	50	50	1	100	100	1	2		>8	>4/76

*: The minimum inhibitory concentration (MIC) of the antibiotics was measured using the BD, Phoenix™ identification and anti-biogram device.

**TABLE 11:** MIC and MFC of the extracts of *R. montana*, and MIC (µg/mL) of antifungals against the Fungal strains studied.

Fungal strains	E (1)			E (2)			E (3)			EO			Antifungals^#^
	CMI	CMF	CMF/CMI	CMI	CMF	CMF/CMI	CMI	CMF	CMF/CMI	CMI	CMF	CMF/CMI	Terbinafine
*C. albicans*	50	50	1	50	50	1	>50	>50	—	9.38	18.75	2	12.500
*C. dubliniensis*	50	50	1	50	50	1	50	50	1	18.75	18.75	1	3.125
*C. tropicalis*	25	25	1	>50	>50	—	50	50	1	9.38	9.38	1	12.500
*C. parapsilosis*	50	50	1	50	50	1	50	50	1	18.75	37.5	2	6.250
*Aspergillus niger*	6.25	6.25	1	3.13	3.13	1	12.5	12.5	1	2.34	4.69	2	3.125

^#^: The MIC, of terbinafine was calculated on a microplate.

According to the results of the antifungal activity illustrated in Table 10, the hydromethanolic extract and the EO of *R. montana* tested against the four *Candida* strains and *Aspergillus niger* revealed promising antifungal activity, while the decoction and hydroethanolic extract showed moderate activity.

For the three extracts, the MIC and MFC values ranged from 3.13 to 50 mg/mL and varied between different fungi; in some cases, they were equal, indicating a fungicidal action. *Aspergillus niger* was the most sensitive, with MIC and MFC values of 12.5, 6.25, and 3.13 mg/mL for the decoction, hydromethanolic extract, and hydroethanolic extract, respectively. However, against the *Candida* yeasts, the decoction and hydroethanolic extract exhibited moderate action, with MIC and MFC values of 50 mg/mL, except for *Candida albicans* and *Candida tropicalis*, where the activity of both extracts was null.

The hydromethanolic extract also exhibited both fungistatic and fungicidal powers against *Candida* yeasts at the same concentration of 50 mg/mL, except for *C. tropicalis*, for which the MIC and MFC were 25 mg/mL.

The essential oil EO, on the other hand, demonstrated a stronger fungicidal effect on the yeasts than the tested extracts. The MIC and MFC values ranged from 2.34 to 37.75 mg/mL. The minimum fungicidal concentration of 4.69 mg/mL was achieved against *A. niger*. The highest MFC of 37.5 mg/mL was recorded against *Candida parapsilosis*. *Candida tropicalis* was inhibited and destroyed at the same concentration of 9.38 mg/mL, while *C. albicans* and *Candida dubliniensis* showed MFC values of 18.75 mg/mL.

For the antibacterial activity, the results obtained from Table 11 show that the extracts and the EO of *R. montana* exhibit activity against bacterial strains. Furthermore, the determination of the MIC of the extracts and EO revealed variable levels of action. Among the three extracts tested, the aqueous ethanolic extract was more active compared to the aqueous methanolic extract and the decoction. The latter was found to be inactive against all the tested bacteria, except for *E. cloacae* and *S. epidermidis*, for which the MIC and MBC were 50 mg/mL.

The methanolic and aqueous ethanolic extracts exhibited both bacteriostatic and bactericidal actions at a concentration of 25 mg/mL against *E. cloacae* and *S. aureus*. Additionally, both extracts showed bactericidal activity at 50 mg/mL against *S. epidermidis*. Moreover, the hydroethanolic extract demonstrated bactericidal activity at 50 mg/mL against the *Klebsiella pneumoniae* strain, while the aqueous methanolic extract was inactive.

Thus, the three extracts tested did not exhibit any effect on wild *Escherichia coli*. In contrast, the essential oil inhibited and destroyed the strains *Enter. cloacae*, *Kleb. pneumoniae*, and wild *E. coli* at a concentration of 50 mg/mL. *S. aureus* and *S. epidermidis* appeared less sensitive, showing higher MIC and MBC values of 100 mg/mL.

The variation in sensitivity of the tested microorganisms to the extracts of *R. montana* could be explained by the distinct composition of secondary metabolites in these extracts. On the other hand, it may be due to the absence of strongly antimicrobial molecules or the low potency of the antibacterial compounds present in the extracts.

The results of the antimicrobial activity of the EO of *R. montana* obtained are consistent with those of Drioiche et al. (2020b). These authors described the antibacterial activity of the EO from *R. montana* from Morocco, primarily containing 2-undecanone (82.62%), as moderate against *Staphylococcus epidermidis*, *K. pneumoniae* spp. pneumoniae, *E. coli*, *Enterobacter cloacae*, and *Staphylococcus aureus* STAIML/MRS/mecA/HLMUP/BLACT. However, the same study revealed a significant antifungal potential of this EO against eight yeasts (*C. albicans*, *C. tropicalis*, *Candida glabrata*, *C. dubliniensis*, *Candida* sp., *Rhodotorula rubra*, *Trichosporon* sp., *Cryptococcus neoformans*), three molds (*A. niger*, *Fusarium* sp., *Penicillium* sp.), and one dermatophyte (*Trichophyton mentagrophytes*), with MFC values of 1.8 mg/mL for yeasts, 0.9 mg/mL for molds, and 0.45 mg/mL for dermatophytes, respectively.

Benali et al. (2020a) reported the moderate antimicrobial action of the EO of *R. montana* from Morocco, predominantly composed of 2-undecanone (63.97%), against eight microbial strains, including *Bacillus subtilis*, *S. aureus*, *E. coli*, *Pseudomonas aeruginosa*, and *C. albicans*, with inhibition zone diameters ranging from 9.2 ± 0.5 mm to 18 mm. Flouchi et al. (2024) also revealed the modest antibacterial activity of the Moroccan *R. montana* EO, which contained 2-undecanone (85.76%), 2-nonanone (3.95%), 2-decanone (3.67%), and 2-dodecanone (1.94%), with MIC values ranging from 4% (v/v) for *Pantoea* to 8% (v/v) for *S. aureus*, *P. aeruginosa*, *E. coli*, *Pantoea* spp., *K. pneumoniae*, *Escherichia hermannii*, *Stenotrophomonas maltophilia*, except for *K. pneumoniae*, where no inhibition was detected.

Another study evaluated the antimicrobial activity of the EO of *R. montana* isolated from the aerial parts collected in Algeria on eight microbial strains (*S. aureus*, *B. subtilis*, *Enterococcus faecium*, *E. coli*, *Klebsiella pneumonia*, and *P. aeruginosa*) and two yeasts (*C. albicans* and *Saccharomyces cerevisiae*) using the agar disc diffusion method. The authors described the antimicrobial activity of this plant as moderate (inhibition zone diameters ≤ 18 mm) (Mohammedi et al., 2019).

Another study compared, using the agar disc diffusion method, the antibacterial activity of *R. montana* EO alone and in combination with five conventional antibiotics (gentamicin, amoxicillin, cefazolin, tetracycline, and amoxicillin/clavulanic acid against three pathogenic bacteria (*S. aureus*, *P. aeruginosa*, and *E. coli*). The results showed that the EO of *R. montana* from Algeria, whose main compounds are 2-undecanone (63.39%), 2-nonanone (5.65%), 2-acetoxytetradecane (4.94%), 2-decanone (4.47%), and 2-dodecanone (3.35%), had no or very weak antibacterial activity against *E. coli* and *S. aureus*, with MIC values ranging from 125 to 250 mg/mL. However, the combination of *R. montana* EO with antibiotics, particularly with amoxicillin and cefazolin, induced significant synergistic effects against all the bacterial strains tested. The inhibition zones for amoxicillin and cefazolin were 19.7–34 mm and 21.5–41 mm, respectively (Zeraib et al., 2021).

Generally, the antimicrobial power of EOs is related to their chemical composition. Some previous studies have reported the moderate antimicrobial activity of EOs from the *Ruta* genus (Coimbra et al., 2020; Nahar et al., 2021). In our study, the modest antimicrobial activity of *R. montana* L. EO could be attributed to the high percentage of aliphatic ketones, which represent 88.6% of the total chemical compounds, with the main compound being 2-undecanone (81.16%). Indeed, the antibacterial activity of this compound is known to be limited against bacterial strains (Gibka et al., 2009; Kunicka-Styczyńska and Gibka, 2010). On the other hand, this low activity could also be due to the multi-resistance of the strains.

### 3.11 Anti-inflammatory activity of *R. montana* extracts and essential oil

This experiment was conducted to determine the ability of the aqueous extract and the EO to reduce inflammation following carrageenan injections to induce inflammatory pain. Starting from the third hour after the carrageenan injection, doses of the aqueous extract and EO of R. montana (100 and 300 mg/kg, 0.1 and 0.2 mL) showed a significant suppression (*p < 0.001*) of paw edema compared to the negative control group (Table 12).

**TABLE 12:** Anti-edematous Effect of the Aqueous Extract E (3) and EO of the Flowering Tops of *R. montana* and Indomethacin (*p < 0.05; **p < 0.01; and ***p < 0.001).

Diameter (cm) and % of inhibition						
Treatment	Dose (mg/kg)	0 h	3 h	4 h	5 h	6 h
Control (NaCl 0.9%)	—	2.36 ± 0.14	2.93 ± 0.08**	2.90 ± 0.05**	2.83 ± 0.03**	2.73 ± 0.03*
Control (corn oil)	—	2.36 ± 0.04	2.83 ± 0.03***	2.83 ± 0.04***	2.73 ± 0.03**	2.63 ± 0.03**
Indomethacine	10	2.33 ± 0.03	2.53 ± 0.03** 65%	2.47 ± 0.01* 75%	2.41 ± 0.01 83%	2.37 ± 0.03 90%
Aqueous extract E (3)	100	2.16 ± 0.03	2.66 ± 0.03*** 13%	2.61 ± 0.03*** 17%	2.53 ± 0.01*** 22%	2.43 ± 0.03*** 28%
	300	2.23 ± 0.03	2.60 ± 0.05** 36%	2.50 ± 0.05* 50%	2.40 ± 0.05 64%	2.31 ± 0.04 79%
EO	0.1 mL	2.20 ± 0.05	2.65 ± 0.02*** 5%	2.56 ± 0.04*** 24%	2.48 ± 0.04** 25%	2.41 ± 0.04* 23%
	0.2 mL	2.30 ± 0.05	2.70 ± 0.02*** 15%	2.58 ± 0.03** 59%	2.48 ± 0.04* 62%	2.38 ± 0.04 71%

However, only the highest concentrations of the aqueous extract and the essential oil (EO) significantly reduced the edema induced by the subplantar injection of carrageenan (*p < 0.001* at 6 h). Indeed, the dose of 300 mg/kg of the aqueous extract and the volume of 0.2 mL of the EO provided the greatest protection against paw volume increases, with suppression values of 79% and 71%, respectively, at the sixth hour. In contrast, the doses of 100 mg/kg of the aqueous extract and the volume of 0.2 mL of the EO showed lower protection compared to the 300 mg/kg dose of the aqueous extract and 0.1 mL of the EO, with suppression values of 28% and 23%, respectively. This indicates that the effectiveness of the anti-inflammatory effects of the aqueous extract and EO of *R. montana* is dose-dependent, and injections at these higher doses are more effective as anti-inflammatory treatments.

When comparing the groups that received indomethacin at a dose of 10 mg/kg, a protective effect of 90% was observed. Although the anti-inflammatory effect of the aqueous extract at a dose of 300 mg/kg and the EO at a dose of 0.2 mL was lower than that of indomethacin (10 mg/kg), both showed comparable anti-inflammatory effects throughout the observation period. Furthermore, after 3 h, the EO and aqueous extract of *R. montana*, as well as the standard medication, showed significant anti-inflammatory efficacy. This could be attributed to the time required for both substances to reach their site of action.

Carrageenan is a phlogistic agent known for its classic biphasic effect. Its administration triggers an inflammatory process, characterized by pain, heat, redness, and especially by swelling of the rat’s hind paw, i.e., edema. The first phase is vascular and lasts between 90 and 180 min. It is characterized by the release of several mediators such as histamine and serotonin. In contrast, the second swelling phase, known as the cellular phase, extends from 270 to 360 min. It is mediated by the release of substances such as prostaglandins and cytokines (TNF-α and IL-1β) (Di Rosa et al., 1971), during which various cells, including neutrophils and monocytes, infiltrate the inflammatory site (Chen et al., 2018).

There is limited scientific information regarding the anti-inflammatory activity of *R. montana* L. Previous studies have described the anti-inflammatory effects of certain *Ruta* species. For example, the methanolic extract of *R. graveolens* leaves collected in India (400 mg/kg) showed a significant inhibitory effect on edema formation after carrageenan injection, with an inhibition percentage of 47.19% after 5 h, which is lower than our results (Kataki et al., 2014). Another group of researchers found that, in an acute model induced by carrageenan, the anti-inflammatory activity of the alkaloid fraction of Indian *R. graveolens* at a dose of 10 mg/kg exhibited superior anti-inflammatory activity (83%) compared to polyphenols (64%) and the standard drug used, diclofenac (70%) (Ratheesh et al., 2009). Another study suggested that the anti-inflammatory activity observed in the ethanolic extract of *R. chalepensis* could be attributed to the presence of flavonoids (Iauk et al., 2004).

Flavonoids, due to their structure, can exert effective anti-inflammatory activity by inhibiting the secretion of factors responsible for inflammation, such as histamine, prostaglandins, serotonin, and lipoxygenases (Oubihi et al., 2020; Abou Baker, 2022; Al-Khayri et al., 2022). Another study revealed that, in the presence of polyphenols, the production of prostaglandins, which are responsible for the onset of inflammation, is reduced (Yahfoufi et al., 2018). HPLC chromatographic analysis of plant extracts showed the presence of the following phenolic compounds: rhamnetin 3-rhamnoside, rosmarinic acid glucoside, quercitrin, p-coumaroylquinic acid, 4-di-O-galloylquinic acid, ferulic acid, and embelin (6.12%). The anti-inflammatory activity of these compounds, such as phenolic acids (Liu et al., 2023), flavonoids (Shahidi and Yeo, 2018), and benzoquinones (Martínez and Bermejo Benito, 2005; Basha et al., 2022), has already been demonstrated in previous studies.

Regarding the EO of *R. montana*, no previous studies have investigated its anti-inflammatory effect. However, prior research has reported the anti-inflammatory activity of EOs from certain *Ruta* species. The EO of *R. chalepensis* from Algeria, composed of 2-undecanone (35.51%), 1-decanol-2-methyl (8.62%), and 2-dodecanone (6.86%), significantly reduced carrageenan-induced edema. The researchers suggested that the anti-inflammatory effect of this essential oil results from the inhibition of inflammation mediators such as serotonin, prostaglandins, and histamine (Boudjema et al., 2018). For the EO of *R. graveolens*, Nonnenmacher et al. (2016) (Nonnenmacher et al., 2016) found that its oral administration at a dose of 100 mg/kg was effective in reducing the edema index and inflammatory process in mice.

The anti-inflammatory effect of the EO of *R. montana* observed in this study is primarily associated with its chemical composition. An *in vivo* study demonstrated that the EO of *Houttuynia cordata* Thunb., of which 2-undecanone is the major component, possesses anti-inflammatory activity. The study showed that 2-undecanone inhibits the formation of ear edema induced by xylene in a dose-dependent manner. Furthermore, its inhibition percentage of ear swelling is similar to that of aspirin at a dose of 8 mg/20 g, reaching 28% (Chen et al., 2014). Another study by Chen et al. (2014) also confirmed the ability of 2-undecanone to reduce xylene-induced ear edema. Li et al. (2011) reported that 2-undecanone inhibits, in a dose-dependent manner, the induction of TNF-α, NO, and H2O2 production by lipopolysaccharides. Therefore, the anti-inflammatory activity of the EO of *R. montana* observed in this study can be attributed to its predominant component, 2-undecanone (81.16%).

### 3.12 Antinociceptive activity of *R. montana*

The abdominal writhing test induced by acetic acid, widely used for its high sensitivity, allows the detection of substances with analgesic effects. In this study, this experimental model is used to determine the analgesic effect of the aqueous extract and essential oil of *R. montana*. Table 13 illustrates the analgesic activity in mice treated with the essential oil and aqueous extract of *R. montana* as well as the reference analgesic, Tramadol (10 mg/kg).

**TABLE 13:** Antinociceptive effect of the aqueous extract and EO of *R. montana* flowering tops and tramadol.

Treatment	Dose	Number of writhes	Writhes inhibition (%)
Negative control (NaCl)		68.67 ± 2.028	
Negative control (Corn oil)		71.67 ± 2.186	
Tramadol	10 mg//kg	15.67 ± 1.764	77.032%
*R. montana*			
Aqueous extract E (3)	100 mg/kg	60.67 ± 1.453	11.621%
	300 mg/kg	52.67 ± 2.404	23.371%
EO	0.1 mL	41.00 ± 1.732	42.820%
	0.2 mL	39.67 ± 0.882	44.554%

The large number of abdominal writhings observed after the injection of acetic acid in the presence of distilled water reveals the algogenic effect of this chemical compound. All tested doses of the aqueous extract and essential oil of *R. montana* significantly reduced (*p < 0.001*) the abdominal writhings induced by acetic acid in rats compared to the negative control groups. A preliminary analysis of the results allowed us to conclude that the aqueous extract and essential oil of this plant exert an analgesic effect, the intensity of which varies depending on the dose administered. The aqueous extract (100 and 300 mg/kg), administered orally 1 h before the injection of the stimulus, significantly (*p < 0.001*) and progressively reduced the writhings of rats exposed to acetic acid (11.621%–23.371%). The analgesic activity of *R. montana* essential oil was found to be superior to that of the aqueous extract. Indeed, the maximal inhibition was achieved with a volume of 0.2 mL (44.554%), which remained lower than that of Tramadol (77.032%). We also observed that a 0.1 mL volume of the essential oil of this plant produced an analgesic effect close (42.820%) to that of 0.2 mL of the EO.

The abdominal writhings induced by acetic acid result from the activation of local peritoneal receptors (Ouédraogo et al., 2012), as well as the release of mediators such as prostaglandins (PGE2α, PGF2α) and cytokines (TNF-α, IL-1β, IL-8) (Bentley et al., 1983; Negus et al., 2006). The analgesic effect expressed by the aqueous extract and essential oil of *R. montana* L. indicates the presence of compounds capable of inhibiting the release of these chemical mediators.

In the literature, there is limited scientific data regarding the analgesic properties of *Ruta* species. The hexane extract of *R. graveolens*, using the acute tail immersion model, revealed a high analgesic effect of 62.1% (Micael et al., 2015). Similarly, the methanolic leaf extract of this species (100 mg/kg) significantly reduced by 54% the number of writhings induced by acetic acid (Loonat and Amabeoku, 2014). These researchers attributed this effect to the secondary metabolites present, such as flavonoids, tannins, saponins, and triterpenoid steroids.

Moreover, previous studies have demonstrated the analgesic effects of tannins, phenolic acids, and flavonoids (Sengupta et al., 2012; Ferraz et al., 2020; Zouhri et al., 2023). Therefore, the effect of our extract could be attributed to its richness in bioactive compounds, primarily phenolic acids and flavonoids.

Regarding essential oils, previous work suggests that their analgesic effects are due to their main constituents and a possible synergy between these chemical components. Their antinociceptive activity relies on peripheral (anti-inflammatory) and/or central mechanisms (Sarmento-Neto et al., 2016).

### 3.13 Subacute toxicity of the essential oil and aqueous extract of *R. montana*

*R. montana*, a plant native to the Mediterranean region, has a long history of medicinal use and is known for its various pharmaceutical activities due to the presence of bioactive compounds. This species contains a significant amount of alkaloids and furocoumarins (Milesi, 2001). Furthermore, ethnobotanical studies have reported cases of poisoning by *R. montana*, manifested by respiratory diseases, skin toxicity, digestive disturbances, and neurological issues (Mitra et al., 2020; Belhaj et al., 2021). In this context, we investigated the subacute toxicity of the essential oil and aqueous extract at doses previously tested for their anti-inflammatory and analgesic activities. The variation in the relative organ weights, as well as biochemical analyses, highlighted the consequences of oral treatments with the essential oil (at 0.1 and 0.2 mL) and the aqueous extract of *R. montana* L. (at 100 and 300 mg/kg) on renal and hepatic functions.

#### 3.13.1 Relative organ weight

The weight of the organs is an important indicator of physiological and pathological states in animals. Table 14 presents the effect of the aqueous extract and essential oil of *R. montana* L. on the relative weight (%) of several organs (kidneys, livers, and spleens) in rats exposed to different doses, compared to the control groups.

**TABLE 14:** The relative mass of the organs of the control and treated rats under the conditions of subacute toxicity by aqueous extracts E (3) and EO of *R. montana*.

Organs	Control NaCl	Control corn oil	E (3) 100 mg/kg	E (3) 300 mg/kg	EO 0.1 mL	EO 0.2 mL
Liver	4.242 ± 0.089	4.137 ± 0.086	3,475 ± 0,269^ns^	3,701 ± 0,206^ns^	4,641 ± 0.075 ^ns^	4,591 ± 0,241^ns^
Kidney	0.787 ± 0.019	0.768 ± 0.034	0.708 ± 0.146	0.670 ± 0.122	0.751 ± 0.028	0.669 ± 0.025
Spleen	0.296 ± 0.005	0.313 ± 0.029	0.301 ± 0.057	0.271 ± 0.052	0.356 ± 0.008	0.306 ± 0.018

The essential oil and aqueous extract, administered at the tested doses to male mice on a daily basis, caused no fatalities or toxic symptoms in the rats. They behaved normally throughout the study and survived until the end of the experiment (28 days). Furthermore, no visible changes in the internal organs were detected macroscopically in any of the rats. The relative organ weight of the livers, kidneys, and spleens of all the tested groups at all tested doses remained within the normal range of the control group.

#### 3.13.2 Study of serum biochemical parameters

The results of the biochemical parameters related to renal and hepatic functions are summarized in Table 15.

**TABLE 15:** Biochemical parameters of rats treated orally with aqueous extract E (3) and essential oil (EO) from *R. montana* for 28 days.

Treatment	Dose	UREA	CREA	ALT	AST
Control (NaCl)	----	0.20 ± 0.01	1.61 ± 0.19	55.33 ± 2.02	82.67 ± 4.63
Control (corn oi)	----	0.24 ± 0.01	2.08 ± 0.16	58.33 ± 4.63	81.00 ± 6.50
Aqueous extract E (3)	100 mg/kg	0.25 ± 0.02	2.36 ± 0.21	49.00 ± 2.64	85.33 ± 2.02
	300 mg/kg	0.25 ± 0.01	1.94 ± 0.26	46.33 ± 3.48	91.33 ± 4.63
EO	0.1 mL	0.26 ± 0.00	1.49 ± 0.12	46.33 ± 2.40	81.33 ± 4.63
	0.2 mL	0.24 ± 0.01	1.50 ± 0.13	47.33 ± 0.88	89.33 ± 0.66

**FIGURE 9:**
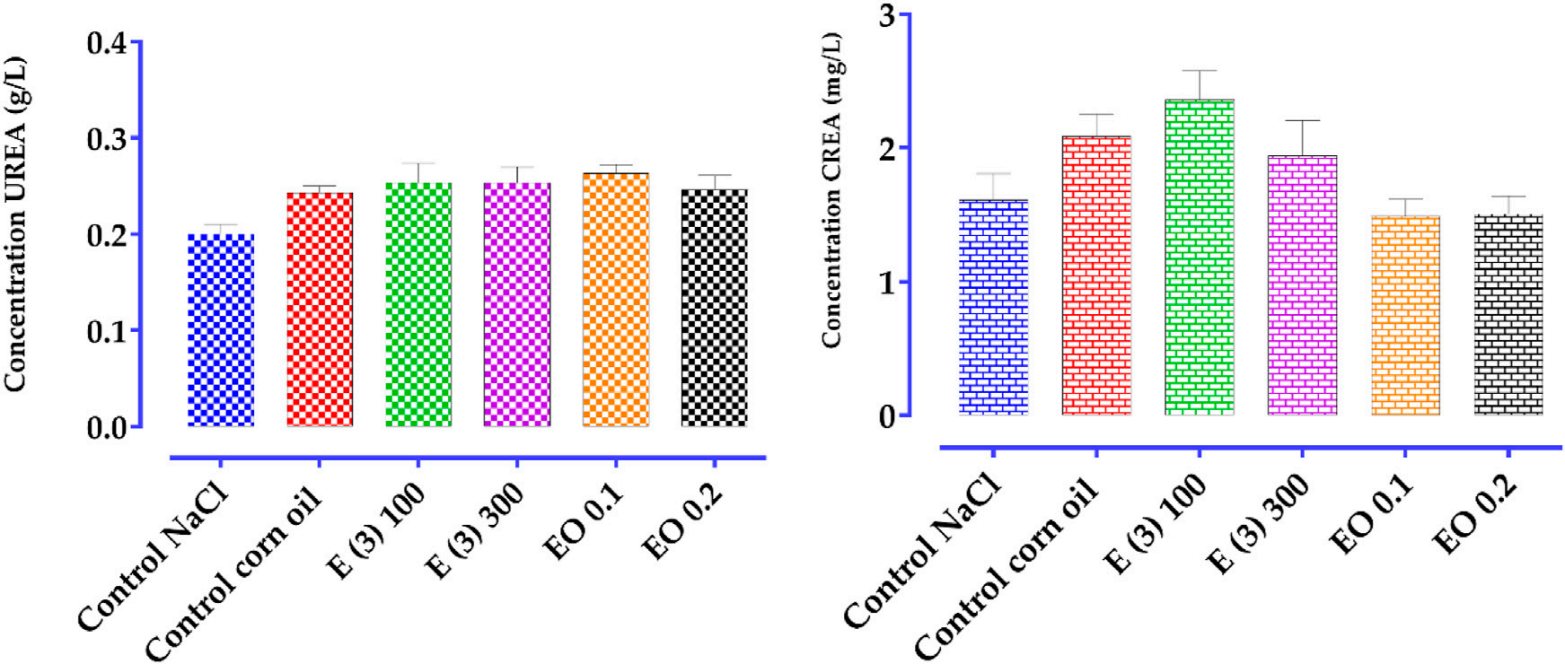
Effect of *R. montana* aqueous extract E (3) and EO on creatinine and urea levels in rats. Values are expressed as mean ± SEM (n = 6). The means display a significant difference (*p < 0.01*).

Many researchers use the liver and kidneys of rats to assess the safety or toxicity of drugs and plant-derived substances (Satyapal et al., 2008). Urea and creatinine are among the main markers of renal function (Treacy et al., 2019). In this study, the biochemical parameters used to evaluate renal function are presented in Figure 9. The daily oral administration of essential oil and aqueous extract of *R. montana* L. did not significantly alter the mean serum levels of uric acid in the experimental rats compared to those of the control rats. These mean values ranged between 0.20 mg/dL and 0.24 mg/dL in the control group and between 0.24 mg/dL and 0.26 mg/dL in the experimental group.

In contrast, creatinine levels slightly increased in a non-significant manner in rats treated with the aqueous extract compared to the control group. These mean values ranged from 1.61 mg/dL to 2.08 mg/dL in the control group, and from 2.36 mg/dL to 1.94 mg/dL, respectively, for the doses of 100 and 300 mg/kg in the experimental group. A slight decrease in creatinine levels was observed in the group of rats treated with 0.1 and 0.3 mL of the essential oil. These results suggest that the essential oil of *R. montana* does not have a negative effect but appears to have a protective effect on the kidneys.

**FIGURE 10:**
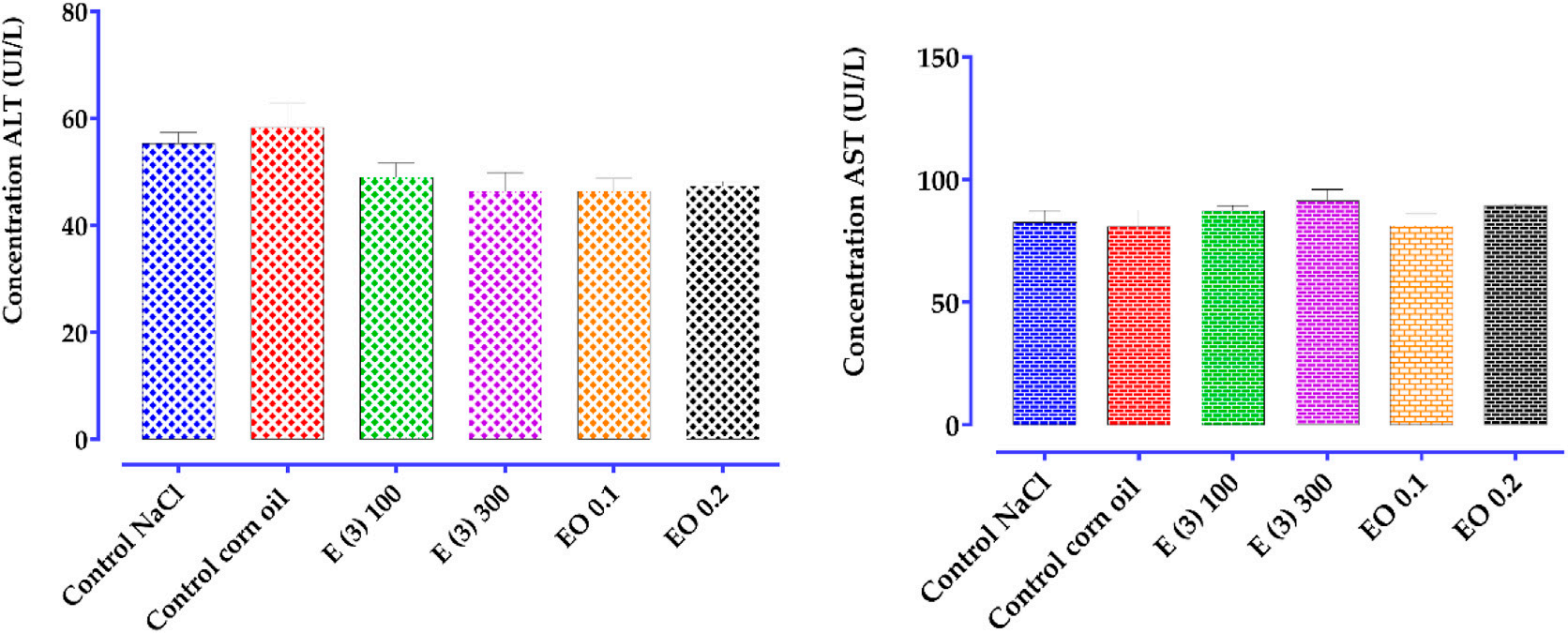
Effect of administration of total aqueous extract E (3) and EO of *R. montana* on some biochemical parameters for the evaluation of hepatic function (AST, ALT) in rats. Values are expressed as mean ± SEM (n = 6). The means display a significant difference (*p < 0.01*).

For liver function, the liver is one of the organs where a large number of compounds derived from plants are accumulated and detoxified (Guan and He, 2015). The analysis of transaminase levels is a good indicator of the toxic effects of medicinal plants on the liver (Adeneye et al., 2006; Amang et al., 2020). In this regard, the potential hepatotoxicity of the extract and essential oil of *R. montana* was evaluated by measuring the enzymatic activities of aminotransferases (ALT and AST). AST (aspartate aminotransferase) is an enzyme known for its rapid response to acute liver damage (within 24 h), with an increase that can reach up to ten times (Whitehead et al., 1999; Al-Busafi and Hilzenrat, 2013). While ALT (alanine aminotransferase) is a highly specific indicator of liver cell damage (Ozer et al., 2008; Adewale et al., 2016). The results of the biochemical parameters analyzed for liver function (ALT and AST) are presented in Figure 10. A slight, non-significant decrease in ALT levels was observed in the rat groups treated with the aqueous extract and essential oil compared to the control group.

For AST, a non-significant increase was detected in the group of rats treated with 100 and 300 mg/kg of the aqueous extract compared to the control group. These mean values ranged from 82.67 UI/L to 81.00 UI/L in the control group, and from 85.33 UI/L to 91.33 UI/L in the experimental group. Similarly, the essential oil at a dose of 0.2 mL induced a non-significant increase in AST levels (89.33 UI/L), while the 0.1 mL dose did not alter the AST levels (81.33 UI/L).

Based on these results, it is reasonable to assume that repeated administration of the aqueous extract at doses of 100 and 300 mg/kg, as well as the essential oil at a dose of 0.2 mL for 28 days, may induce toxicity in vital organs such as the heart and kidneys. In contrast, the essential oil at a dose of 0.1 mL appears to have hepatoprotective and nephroprotective effects.

## 4 Conclusion

This study provided a detailed chemical characterization and a comprehensive evaluation of the biological properties of *Ruta montana L.* from Morocco, highlighting its therapeutic potential. The essential oil, predominantly composed of 2-undecanone (81.16%) and decyl propanoate (9.33%), exhibited significant antimicrobial activity, particularly against *Candida albicans*, *Candida tropicalis*, and *Aspergillus niger*.

The phenolic extracts, enriched with compounds such as rosmarinic acid 3′-glucoside and quercitrin, demonstrated notable antioxidant capacity. Furthermore, anti-inflammatory and analgesic assays revealed dose-dependent efficacy, with the essential oil showing effects comparable to standard treatments like indomethacin. Notably, analgesic tests indicated a marked reduction in pain induced by acetic acid when using the essential oil.

From a toxicological perspective, the essential oil and extracts were found to be relatively safe at controlled doses, although slight increases in AST and creatinine levels at higher concentrations warrant further investigation.

*R. montana* presents promising prospects for the development of natural antimicrobial agents, antioxidant supplements, and treatments for inflammation and pain. Further clinical studies, long-term safety evaluations, and exploration of interactions with other bioactive compounds are essential to fully harness its pharmaceutical and nutraceutical potential.

## Data Availability

The raw data supporting the conclusions of this article will be made available by the authors, without undue reservation.
