# Supplementary material for: Ruta montana L. from Morocco: comprehensive phytochemical analysis and exploration of its antioxidant, antimicrobial, anti-inflammatory and analgesic properties
Source: Front Chem. 2025 Jun 20;13:1614984. doi: 10.3389/fchem.2025.1614984 (PMC12278426; doi:10.3389/fchem.2025.1614984)
Supplement: Supplementary file 1 [file DataSheet1.docx]

[Supplementary Table S1](https://www.frontiersin.org/journals/chemistry/articles/10.3389/fchem.2019.00642/full#SM1). *R. montana* origin, parts used, habitat, and season of harvest.

| ***Scientific name*** | **Part**  **Collected** | **Harvesting Area** | | | | | |
| --- | --- | --- | --- | --- | --- | --- | --- |
|  |  | **Region** | **Locality** | **Latitude (x)** | **Longitude (y)** | **Altitude (m)** | **Harvesting time** |
| *Ruta montana* L. | Flowering tops | Bouelamne | Guigo | 33° 21′ 55″N | 4° 49′ 34″ W | 1748m | June 2024 |

[Supplementary Table S2](https://www.frontiersin.org/journals/chemistry/articles/10.3389/fchem.2019.00642/full#SM1)**.** Coding of *R. montana* Extracts

| **Extraction Method** | **Extracts** | **Coding** |
| --- | --- | --- |
| **Soxhlet** | Hydro-methanolic extract | E (1) |
|  | Hydro-ethanolic extract | E (2) |
| **Decoction** | Aqueous extract | E (3) |


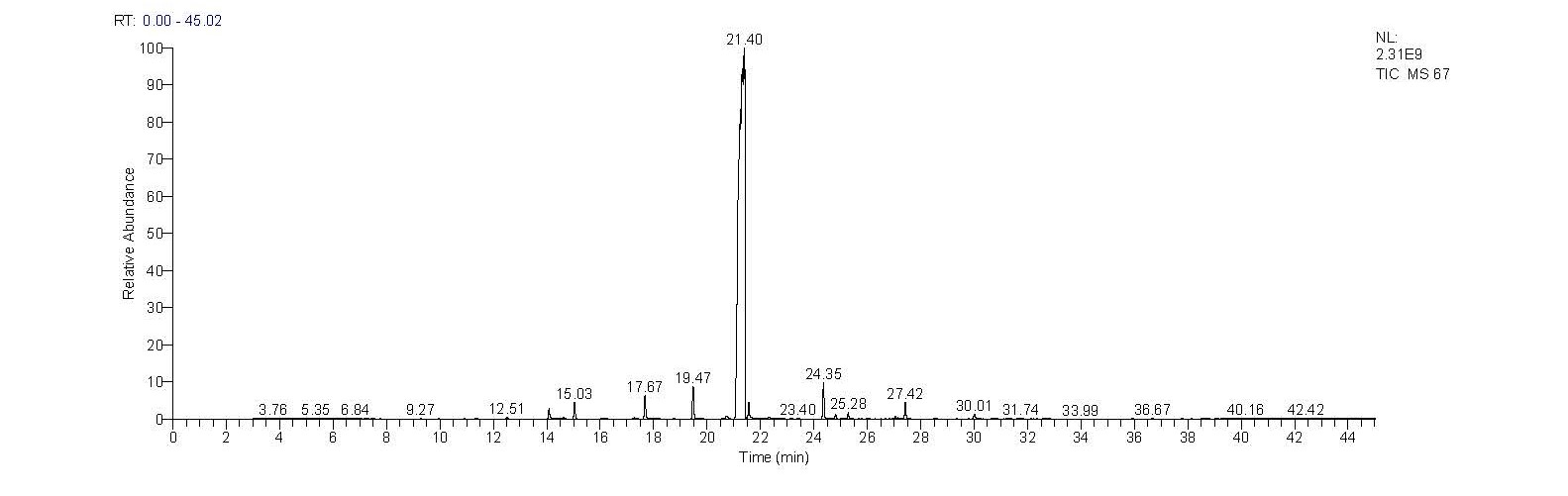


[Supplementary Figure S1](https://www.frontiersin.org/journals/chemistry/articles/10.3389/fchem.2019.00642/full#SM1) **.** Chromatogram of the essential oil from *R. montana*
